# On the precision models of fringe projection profilometry: unification, simplification and connection

**DOI:** 10.1038/s41377-026-02300-x

**Published:** 2026-05-12

**Authors:** Shenzhen Lv, Nengqi Huang, Yuxuan Zou, Chengeng Liu, Siah Yee Long, Dawei Tang, Xiangqian Jiang, Qian Kemao

**Affiliations:** 1 College of Computing and Data Science, Nanyang Technological University, Singapore, Singapore; 2 Centre for Precision Technologies, University of Huddersfield, Huddersfield, UK

**Keywords:** Imaging and sensing, Optical sensors

## Abstract

Fringe projection profilometry (FPP) is one of the most prominent non-interferometric high-precision 3D shape measurement techniques for measuring dimensions ranging from 1 mm to 1 m, with increasing applications in precision engineering. Thorough theoretical understanding of the precision of the prevailing FPP methods is critical when facing the challenges of higher precision demands. In this paper, through theoretical analysis of three reconstruction methods based on the projection of vertical fringes (Ver3), horizontal fringes (Hor3) and optimal-angle fringes (OptE3), we establish a general unified precision model, from which a general Pythagoras relationship is discovered, clarifying the optimal precision of OptE3. We then simplify the model to reveal the role of a system’s geometry for easier configuration. First, a special yet not restrictive case where the direction of the optical axis of the projector is perpendicular to the baseline of the FPP is considered, resulting in an intermediate unified precision model. We show that the effective baselines of the three methods obey a special Pythagoras relationship. Second, the angle between the optical axes of the camera and the projector is interestingly delineated from the projector’s extrinsic parameters, leading to our final and simplified unified precision model. This simplified model is geometrically tangible and thus eases the determination of the positions and orientations of the projector and camera in system configuration, given a precision budget, for which, an FPP-Planner software tool is prototyped. Going beyond, we theoretically equivalate FPP with stereo vision and laser triangulation regarding precision. Our findings are experimentally validated. These theoretical models are believed to potentially enhance or even change the way of FPP design and performance evaluation.

## Graphical abstract

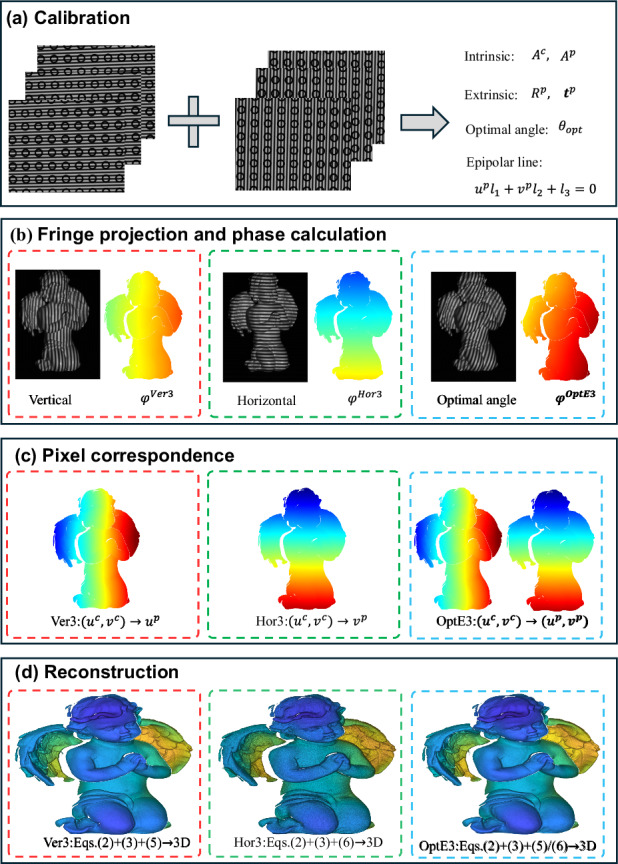

## Introduction

Three-dimensional (3D) shape measurement technologies provide direct and powerful tools for modern intelligent manufacturing^1,2^, autonomous driving^3,4^, medical diagnostics^5,6^, digital documentation of historical landmarks^7,8^ and so on. Due to their low sensitivity to environment, non-interferometric optical approaches have rapidly emerged in innovative applications, such as stereo vision in autonomous driving^9^ and speckle projection in face recognition^10,11^. As yet another 3D measurement technique, fringe projection profilometry (FPP) is distinguished by its high precision (up to several micro-meters) and high speed (kHz and higher), and have gained widespread attention and numerous successful applications in measuring dimensions ranging from 1 mm to 1 m^12–17^.

**Fig. 1:**
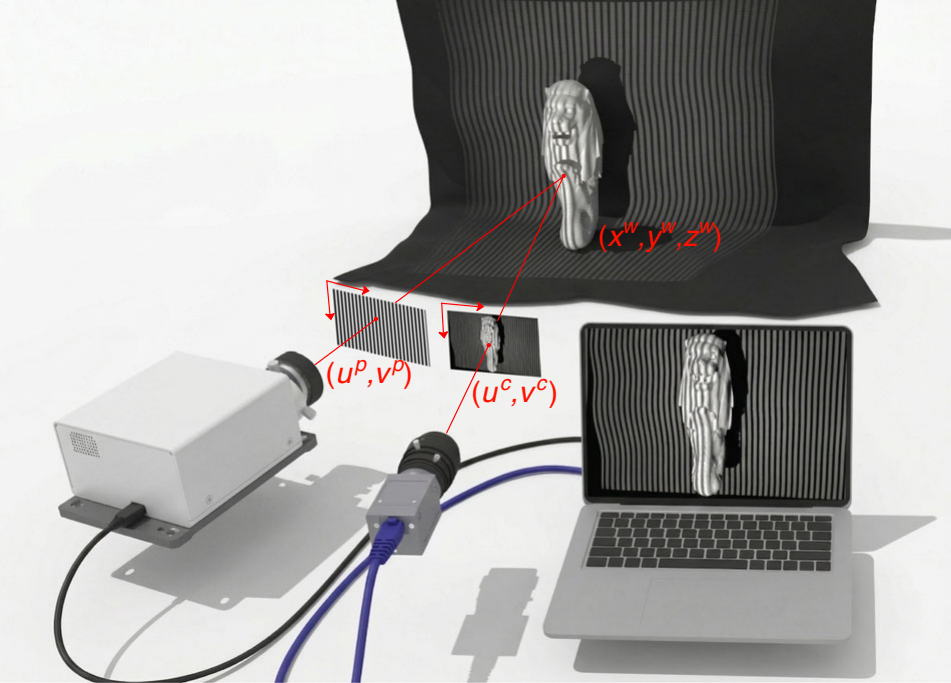
A typical FPP system

A typical FPP system only requires a projector, a camera and a computer. As shown in Fig. 1, straight fringe patterns are projected onto an object, deformed due to the object surface, and captured by a camera. By comparing the straight and deformed patterns, the object shape can be reconstructed. To achieve this, for each camera pixel $\left({u}^{c},{v}^{c}\right)$, a projector pixel $\left({u}^{p},{v}^{p}\right)$ should be found such that they are related to the same 3D object point $\left({x}^{w},{y}^{w},{z}^{w}\right)$, which is feasible as $\left({u}^{c},{v}^{c}\right)$ and $\left({u}^{p},{v}^{p}\right)$ share the same phase value from these fringe patterns. Once a camera pixel and a project pixel are corresponded, each of ${u}^{c},{v}^{c},{u}^{p}$ and ${v}^{p}$ provides a ray-tracing line equation relating to $\left({x}^{w},{y}^{w},{z}^{w}\right)$, resulting in a total of four equations^18–20^. Mathematically, any three of these four equations are sufficient to calculate $\left({x}^{w},{y}^{w},{z}^{w}\right)$.

Due to the flexibility in choosing the fringe orientation for projection and the number of line equations for reconstruction, the following methods have been formed and used in literature noticeably, and will be interrogated throughout this paper:

(i) Ver3: Vertical fringe patterns are projected. For each $\left({u}^{c},{v}^{c}\right)$, its absolute phase is obtained through phase measurement and phase unwrapping^21^. A vertical line of projector pixels with the same phase value is searched, from which, ${u}^{p}$ is determined^22^. The three equations based on ${u}^{c}$, ${v}^{c}$ and ${u}^{p}$ are adequate to reconstruct the 3D object point. This method is called Ver3 for convenience, reflecting the fact of the utilization of vertical fringes for projection and three equations for reconstruction^20^. It is most widely used among others^21^.
(ii) Hor3: This method is similar to Ver3, but projects horizontal fringes instead, and search ${v}^{p}$ to correspond to $\left({u}^{c},{v}^{c}\right)$, and then solves three equations based on ${u}^{c}$, ${v}^{c}$ and ${v}^{p}$ ^20^. Hor3 is less used due to system configuration but has the same theoretical importances as Ver3.
(iii) OptE3: The fringe angles are chosen to be along an optimal angle which is perpendicular to the epipolar line of the system. For each $\left({u}^{c},{v}^{c}\right)$ in the camera, its absolute phase is obtained as in other methods. A line with the same phase value in the projector is found, which is, in general, no longer horizontal or vertical. The intersection point of this line and the epipolar line gives $\left({u}^{p},{v}^{p}\right)$ corresponding to $\left({u}^{c},{v}^{c}\right)$. The four equations based on ${u}^{c}$, ${v}^{c}$, ${u}^{p}$ and ${v}^{p}$ are available for reconstruction, but one is actually redundant thus only three are necessary^20^. The naming of OptE3 indicates the involvement of the *optimal* angle in projection, the epipolar line as a system characteristic, and *three* equations for reconstruction. This method was developed recently, with a unique feature that system configuration (hardware) and optimal fringe angle (algorithm) are tightly connected^20^. Although we were involved in the development of OptE3, we selected this method for study because of its important role to be discovered later in this paper, instead of our personal bias.

Understanding the precision characteristics of each method mentioned above is essential in measurement. For this purpose, we have established a full-chain precision model for Ver3^22^. While this precision model can be readily extended to Hor3, its extension to OptE3 is far from straightforward as OptE3 is more complicated. As a consequence, in our preliminarily precision model of OptE3 presented in ref. ^20^, simplicity was pursued, which unfortunately obscured any deeper connections between OptE3 and Ver3/Hor3. This interesting point will be mentioned in Materials and Methods Therefore, we are strongly motivated to have a clearer understanding of all these three methods and will re-examine their precision models in a more systematic approach. As a result, concrete similarity is revealed, which enables us to unify them into just one model, for the first time.

This unified model enables us to find a general Pythagoras relationship: the reciprocals of precisions of Ver3/Hor3/OptE3 form a precision right-angle triangle (PRT). This relationship is useful in choosing a suitable reconstruction method among others - the popular Ver3 and Hor3 are simpler to use while the recent OptE3 has higher precision under the same geometrical configuration.

The above general unified model is represented by the many intrinsic and extrinsic parameters of the projector and the camera. These parameters are intangible and need to be calibrated. For this reason, two-step model simplification is performed. In the first step of simplification, we restrict ${t}_{3}^{p}=0$, i.e., the optical axis of the projector is perpendicular to the baseline of the FPP, without losing generality. We are then able to simplify the unified model and reveal the geometrical role of different methods. Particularly, effective baseline is defined for Ver3/Hor3/OptE3, which incorporates both focal lengths and geometrical baselines. Interestingly, the PRT will be evolved into an effective baseline right-angle triangle (EBRT). As the longer effective baseline increases precision, EBRT clearly indicates the precision performance of each method. The precision of Ver3 will be shown to be only dependent on ${t}_{1}^{p}$ and independent of ${t}_{2}^{p}$, and conversely, the precision of Hor3 be dependent on ${t}_{2}^{p}$ and independent of ${t}_{1}^{p}$.

In the second step of simplification, we work on the angle $\omega$ between the optical axes of the camera and projector, which is an important parameter that affects the performance of FPP. This angle $\omega$ is embedded in a rotation matrix ${R}^{p}$^18^$,$ but we will delineate it from ${R}^{p}$ when the system hardware is properly selected. This leads to our final and simple practical precision model that is useful for system design with a given error budget. Based on this model, an FPP-Planner is prototyped for precision-aware FPP system design.

Finally, we connect our models to stereo vision and laser triangulation. Since Zhang insightfully regarded the projector as an inverse camera in 2006, FPP has been regarded as stereo vision, which is well known to researchers and practitioners^18^. Since both FPP and stereo vision reconstruct 3D geometry by triangulation, they are expected to perform comparably. This viewpoint is supported in this paper from the equivalence of their quantified precision models. As for laser triangulation, it is similar to FPP in the triangulation-based reconstruction, but the dominant source is speckle^2^. The influence of the angle $\omega$ was noticeably discussed in laser triangulation^23,24^, showing that the distance measurement error is inversely proportional to $\sin ({\rm{\omega }})$. By some theoretical derivations, we show that the precision models for FPP and laser triangulation are consistent, surprisingly and interestingly.

In this paper, we adopt an analysis approach, starting from a comprehensive, general but valid model and simplifying it into a simple one through detailed and lengthy analysis. One may argue that such a final model may be easily obtained from an ideal FPP setup where the positions and orientations of the projector and camera are similar to the ones in our final analysis stage. While this can indeed provide some insights into the precision of FPP, it also gives a gap between theory and practice, since a practical system can never be ideal. We work on the opposite approach, so that a general unified model is obtained first, which is always valid to evaluate the precision of any practical FPP system once calibrated. By setting the clear geometrical condition for simplification, it clarifies the gap between the general and special models, in terms of approximation error. Such simplified model is then more useful to properly support the system design. We believe that our work is valuable in facing the higher precision demand from industry, such as advanced manufacturing^25^ and semiconductor industry^26^. The detailed theoretical analysis presented in this paper can also be extended to FPP systems composed of telecentric lenses, Scheimpflug lenses, and the like. A relative work^27^ establishes an optimization framework to design system parameters for improving accuracy, while our paper focuses on precision.

As this work has a stem from our previous ones^20,22^, we highlight their differences. While ref. ^20^. developed OptE3, this work embraces Ver3/Hor3 into one picture. While ref. ^22^. emphasized the full-chain model of Ver3, this work delves into 3D reconstruction, establishes the relationship between Ver3/Hor3/OptE3, simplifies the result for geometrical insights. Furthermore, this paper moves forward towards system design based on the precision models, and connects FPP to stereo vision and laser triangulation. Thus, this work uniquely unifies FPP precision models, connects the sister methods, and bridges the practical FPP system design and theoretical precision analysis.

**Fig. 2:**
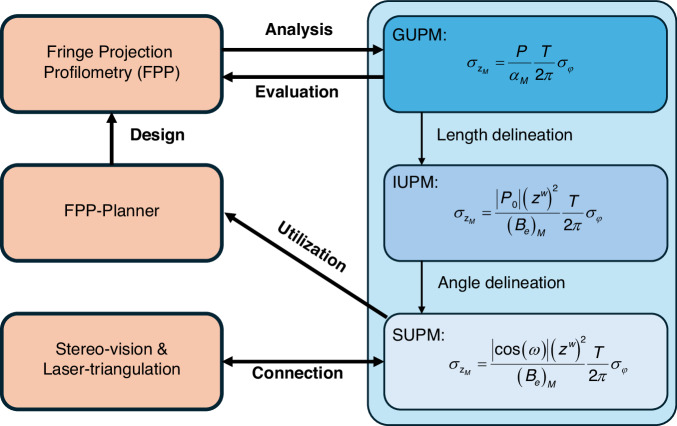
The overall structure of our work. On the right are three precision models derived from FPP, from general (GUPM) to intermediate (IUPM) and simplified (SUPM). On the left, two other optical measurement methods (stereo vision and laser triangulation) are linked up quantitatively and solidly into one family through precision models; in addition, the FPP-Planner links theory and practice into a closed design loop, also through precision models

The overall structure of our work is illustrated in Fig. 2 below, where our main findings are shown as three unified models (general, intermediate and simplified) enabling both pre- and post-evaluation of an FPP system, the FPP-planner making the system design possible, as well as the interesting connections between different optical measurement methods (FPP, stereo vision and laser triangulation) through their measurement precisions. Accordingly, our main contributions are summarized as follows:

(i) A general unified precision model is established for Ver3/Hor3/OptE3, revealing a PRT relationship among them. The model can be used for post-evaluation of an existing FPP system after it is calibrated;
(ii) An intermediate unified precision model is derived under the condition ${t}_{3}^{p}=0$, delineating the geometric length and revealing an EBRT relationship. Furthermore, we analyze how strictly this condition needs to be satisfied in practical systems. This model clearly demonstrates that the differences among Ver3, Hor3 and OptE3 arise from their use of distinct effective baselines;
(iii) A physically simple and intuitive precision model is further derived by delineating and elucidating the role of the optical axis angle. In addition, we systematically evaluate the errors introduced by neglecting the terms on the field of view (FOV) in the model;
(iv) Based on the simplified precision model, we propose a practical system-design strategy and develop the FPP-Planner software, which can directly assist engineers in designing and optimizing FPP systems;
(v) A connection to stereo vision and laser triangulation is drawn through precision quantification, which, interestingly, shows that they share a similar precision characteristic.

## Results

The aim of our research is to provide backbone precision models for different FPP methods (Ver3/Hor3/OptE3), which are achieved through in-depth theoretical analysis. The principle of FPP relies on physics called pinhole model, which is briefly reviewed in Supplementary Note 1. Furthermore, the 4-step FPP workflow of Ver3/Hor3/OptE3, including (i) calibration, (ii) fringe projection and phase computation, (iii) pixel correspondence, and (iv) 3D reconstruction, is summarized in Supplementary Note 1. Our results are then derived as precision model unification, simplification and connection in Parts A, B (including B1, B2 and B3) and C below, respectively. While the simplification may be seen similar to^22^, we highlight that such a process is applied to the unified model, with novel and interesting findings, as will be seen in Part B.

### A: Model unification

In literature, the precision model of Ver3 has been obtained^22^, while that of Hor3 is missing, and that of OptE3 is in a different formulation. By developing the models towards the same style, these different methods, for the first time, are unified as one. The detailed theoretically derivations of these precision models are given in Materials and Methods, with the models for Ver3/Hor3/OptE3 given as Eqs. (37), (38) and (43), respectively. Although complicated, their unexpected similarity can now be easily observed. By a reasonable assumption that ${\sigma }_{{\varphi }^{{Ver}3}}^{2}={\sigma }_{{\varphi }^{{Hor}3}}^{2}={\sigma }_{{\varphi }^{{OptE}3}}^{2}={\sigma }_{\varphi }^{2}$, these models can be expressed in the following general unified precision model (GUPM), which is believed to be fundamental,

1 $${\sigma }_{{z}_{M}}=\frac{P}{{\alpha }_{M}}\frac{T}{2\pi }{{\rm{\sigma }}}_{\varphi }$$

where

2 $${{\rm{\alpha }}}_{M}=\left\{\begin{array}{l}\begin{array}{cc}\left|{Q}_{u}\right|, & \,\,\,\,\,\,\,\,\,\,\,\,\,\,{for\; M}={Ver}3\end{array}\\ \begin{array}{cc}\left|{Q}_{v}\right|, & \,\,\,\,\,\,\,\,\,\,\,\,\,\,{for\; M}={Hor}3\end{array}\\ \begin{array}{cc}\sqrt{{Q}_{u}^{2}+{Q}_{v}^{2}}, & \,\,\,{for\; M}={OptE}3\end{array}\end{array}\right.$$

3 $$P={\left[{r}_{31}^{p}\frac{\left({u}^{c}-{u}_{0}^{c}\right)}{{f}_{u}^{c}}{z}^{w}+{r}_{32}^{p}\frac{\left({v}^{c}-{v}_{0}^{c}\right)}{{f}_{v}^{c}}{z}^{w}+{r}_{33}^{p}{z}^{w}+{t}_{3}^{p}\right]}^{2}$$

4 $${Q}_{u}={f}_{u}^{p}\left[\left({r}_{11}^{p}{t}_{3}^{p}-{t}_{1}^{p}{r}_{31}^{p}\right)\frac{\left({u}^{c}-{u}_{0}^{c}\right)}{{f}_{u}^{c}}+\left({r}_{12}^{p}{t}_{3}^{p}-{t}_{1}^{p}{r}_{32}^{p}\right)\frac{\left({v}^{c}-{v}_{0}^{c}\right)}{{f}_{v}^{c}}+\left({r}_{13}^{p}{t}_{3}^{p}-{t}_{1}^{p}{r}_{33}^{p}\right)\right]$$

5 $${Q}_{v}={f}_{v}^{p}\left[\left({r}_{21}^{p}{t}_{3}^{p}-{t}_{2}^{p}{r}_{31}^{p}\right)\frac{\left({u}^{c}-{u}_{0}^{c}\right)}{{f}_{u}^{c}}+\left({r}_{22}^{p}{t}_{3}^{p}-{t}_{2}^{p}{r}_{32}^{p}\right)\frac{\left({v}^{c}-{v}_{0}^{c}\right)}{{f}_{v}^{c}}+\left({r}_{23}^{p}{t}_{3}^{p}-{t}_{2}^{p}{r}_{33}^{p}\right)\right]$$

Interestingly, ${Q}_{u}$ and ${Q}_{v}$ are not unfamiliar to us - they are the numerator and denominator of the right hand of Eq. (A11) (see Supplementary Note 1), respectively.

Although the above model is still seen complicated, it is interesting to note that the precisions of Ver3/Hor3/OptE3 are only different by a factor ${{\rm{\alpha }}}_{M}$, which reveals the following relationship,

6 $${\sigma }_{{z}_{V{er}3}}:{\sigma }_{{z}_{{Hor}3}}:{\sigma }_{{z}_{{OptE}3}}=\frac{1}{s{in}\left({\theta }_{{opt}}\right)}:\frac{1}{\left|c{os}\left({\theta }_{{opt}}\right)\right|}:1$$

or in another form as

7 $${\left(\frac{1}{{\sigma }_{{z}_{V{er}3}}}\right)}^{2}+{\left(\frac{1}{{\sigma }_{{z}_{{Hor}3}}}\right)}^{2}={\left(\frac{1}{{\sigma }_{{z}_{{OptE}3}}}\right)}^{2}$$

**Fig. 3:**
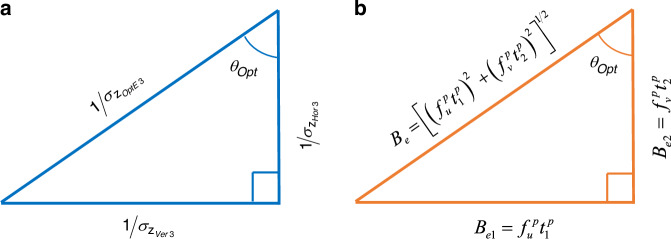
Pythagorean relationships among Ver3/Hor3/OptE3. **a** The general Pythagorean relationship of Ver3, Hor3 and OptE3; **b** The special Pythagorean relationship of effective baseline triangle where ${t}_{1}^{p} > 0$ and ${t}_{2}^{p} > 0$ are assumed. Otherwise, their absolute values should be used

We call Eq. (7) as *general Pythagorean relationship* as illustrated in Fig. 3a, where the triangle is named as precision right-angle triangle (PRT). This result was recently also obtained through a different perspective^28^ and thus theoretically well supported. Figure 3a and Eq. (7) can be seen as a decomposition, but the quantities of the triangle sides do not possess physical meaning by now. The findings and the consequent significances of this unified general model are as follows:

(i) The different methods, Ver3/Hor3/OptE3, can be seen as one, although their respective practical operations are different. Their precisions are related by an interesting general Pythagorean relationship, where OptE3 has the smallest standard deviation and thus highest precision;
(ii) This formula is an identity, independent of the system configuration. Thus, this model is general and fundamental in the sense that it is applicable to arbitrary system configurations;
(iii) As long as an FPP system has been calibrated, its precision can be fully evaluated, which can be used to complement the prevailing experimental evaluation using standard test samples.

### B1: The first model simplification through length delineation

Although general, GUPM is not convenient for practical use and also hides possible physical intuitions, as too many parameters are present. These parameters need to be calibrated and thus difficult to know during system design and before system configuration. Our simplification will move the precision model closer to engineers’ practice, in an explainable and quantified way.

As the first step of simplification, we set ${t}_{3}^{p}=0$. This setting requires the projector’s optical axis to be perpendicular to the line connecting the centers of the camera and projector. This is not immediately apparent, and a detailed proof is provided in Part B2 below. Moreover, setting ${t}_{3}^{p}=0$ implies that the epipole of the projector lies at infinity, in which case the optimal fringe pattern becomes a tilted fringe rather than a circular one^29^. By doing so, we can define a common term of

8 $${P}_{0}={r}_{31}^{p}\frac{\left({u}^{c}-{u}_{0}^{c}\right)}{{f}_{u}^{c}}+{r}_{32}^{p}\frac{\left({v}^{c}-{v}_{0}^{c}\right)}{{f}_{v}^{c}}+{r}_{33}^{p}$$

with which, $P$, ${Q}_{u}$ and ${Q}_{v}$ in Eqs. (3–5) are simplified into $P{\prime}$, ${Q}_{u}^{{\prime} }$ and ${Q}_{v}^{{\prime} }$, respectively, as follows,

9 $${P}^{{\prime} }={\left({z}^{w}\right)}^{2}{P}_{0}^{2}$$

10 $${Q}_{u}^{{\prime} }=-{f}_{u}^{p}{t}_{1}^{p}{P}_{0}$$

11 $${Q}_{v}^{{\prime} }=-{f}_{v}^{p}{t}_{2}^{p}{P}_{0}$$

The optimal angle in Eq. (A11) is accordingly simplified as

12 $$tan({\theta }_{{opt}})=\frac{{Q}_{u}^{{\prime} }}{{Q}_{v}^{{\prime} }}=\frac{{f}_{u}^{p}{t}_{1}^{p}}{{f}_{v}^{p}{t}_{2}^{p}}$$

which is a constant and no longer varies with $\left({u}^{c},{v}^{c}\right)$. This equation enables us to construct another right-angle triangle as shown in Fig. 3(b). While the traditional geometrical baseline is the geometrical distance between two optical centers as

13 $${B}_{0}=\sqrt{{\left({t}_{1}^{p}\right)}^{2}+{\left({t}_{2}^{p}\right)}^{2}}$$

we take the focal lengths into consideration and define the effective baseline as the hypotenuse of the triangle in Fig. 3b,

14 $${B}_{e0}=\sqrt{{\left({f}_{u}^{p}{t}_{1}^{p}\right)}^{2}+{\left({f}_{v}^{p}{t}_{2}^{p}\right)}^{2}}$$

This definition considers the fact that the vertical and horizontal focal lengths as calibration parameters could be different^30^. Nevertheless, in the case that ${f}_{u}^{p}={f}_{v}^{p}=f$, we can return to a simple form that ${B}_{e0}={B}_{0}f$. According to Fig. 3b, ${B}_{e1}{=f}_{u}^{p}{t}_{1}^{p}$ and ${B}_{e2}={f}_{v}^{p}{t}_{2}^{p}$ are simply the vertical and horizontal projections of ${B}_{e}$, respectively. We thus call this triangle as effective baseline right-angle triangle (EBRT), with the following *special Pythagorean relationship*,

15 $${\left({B}_{e1}\right)}^{2}+{\left({B}_{e2}\right)}^{2}={\left({B}_{e0}\right)}^{2}$$

Comparing with the PRT, EBRT shows a clearer physical meaning of decomposition, since it is about effective baselines. Nevertheless, EBRT requires the condition of ${t}_{3}^{p}=0$, which is further discussed in Part A of Discussions.

With these simplified terms in Eqs. (8–11) and the definition of the effective baseline ${B}_{e}$, we can re-write the GUPM in Eq. (1) into an intermediate unified precision model (IUPM) as

16 $${\sigma }_{{Z}_{M}}=\frac{\left|{P}_{0}\right|{\left({z}^{w}\right)}^{2}}{{\left({B}_{e}\right)}_{M}}\frac{T}{2\pi }{\sigma }_{\varphi }$$

where

17 $${\left({B}_{e}\right)}_{M}=\left\{\begin{array}{l}\begin{array}{cc}{B}_{e1}={B}_{e0}s{in}\left({\theta }_{{opt}}\right) & \,\,\,\,\,{for}\,M={Ver}3\end{array}\\ {B}_{e2}={B}_{e0}\begin{array}{cc}\left|c{os}\left({\theta }_{{opt}}\right)\right|\, & {forM}={Hor}3\end{array}\\ \begin{array}{cc}{B}_{e0} & \,\,\,\,\,\,\,\,\,\,\,\,\,\,\,\,\,\,\,\,\,\,\,\,\,{for}\,M={OptE}3\end{array}\end{array}\right.$$

This re-formulation clearly reveals that the standard deviation of Ver3/Hor3/OptE3, i.e., ${\sigma }_{{Z}_{M}}$, is proportional to the object distance, i.e., ${\left({z}^{w}\right)}^{2}$, and inversely proportional to the respective effective baseline, i.e., ${\left({B}_{e}\right)}_{M}$, which makes the theoretical expression more intuitive and practically useful. If allowed in a measurement task, a shorter measurement distance and a longer effective baseline are preferred for a higher precision.

Given a configured FPP system with ${{\boldsymbol{t}}}^{p}=({t}_{1}^{p},{t}_{2}^{p},0)$, the significance of IUPM is as follows:

(i) It reveals the important role of baseline and the interesting special Pythagorean relationship. Particularly, OptE3 has the longest effective baseline ${\left({B}_{e}\right)}_{M}$, and thus has the highest precision. OptE3 utilizes both ${t}_{1}^{p}$ and ${t}_{2}^{p}$, allowing greater freedom for performance improvement, i.e., by increasing ${t}_{1}^{p}$, or ${t}_{2}^{p}$, or both of them;
(ii) For Ver3, its effective baseline ${B}_{e1}$ is the vertical projection of ${B}_{e}$, thus, (a) the effective baseline becomes shorter, and its precision will not exceed that of OptE3; (b) ${B}_{e1}$ only depends on ${t}_{1}^{p}$. If ${t}_{1}^{p}$ approaches 0, terrible results will occur. To increase the precision, ${t}_{1}^{p}$ should be made longer; (c) ${B}_{e1}$ is independent of ${t}_{2}^{p}$, so that even when the projector and/or the camera are moved up and down, the precision remains the same. The points in (b) and (c) have been seen in practice^31,32^, more like an experience. Hor3 is similar to Ver3: (a) its precision does not exceed that of OptE3; (b) ${B}_{e2}$ only depends on ${t}_{2}^{p}$; and (c) ${B}_{e2}$ is independent of ${t}_{1}^{p}$;
(iii) From the reconstruction point of view, Ver3 can be considered as a special case of OptE3 with ${t}_{2}^{p}=0$. We have tested that, when ${f}_{u}^{p}\approx {f}_{v}^{p}$, and ${t}_{1}^{p}/{t}_{2}^{p}\ge 3$, i.e., $\sin \left({\theta }_{{opt}}\right) > 0.95$, then the relative difference between the precisions of OptE3 and Ver3 does not exceed 5%. Similary, Hor3 can be considered as a special case of OptE3 with ${t}_{1}^{p}=0$. Nevertheless, Ver3 and Hor3 have the advantage of simplicity.
(iv) To choose a method for practical use, OptE3 can be considered to fully utilize the effective baseline, especially when a compact FPP system is desired; Ver3 with a sufficiently long ${t}_{1}^{p}$ and Hor3 with a sufficiently long ${t}_{2}^{p}$ are both good options to fulfill the precision requirement, when there is no space constraint. As a remark, during FPP system setup, attention could be paid to the values of ${t}_{1}^{p}$ and ${t}_{2}^{p}$, for better understanding of the system configuration and for possible adjustment.

### B2: The second model simplification through angle delineation

Although the model in Eq. (16) is simpler than that in Eq. (1), the term $\left|{P}_{0}\right|$ yet contains intangible calibration parameters. Interestingly, this term can also be simplified. We introduce the angle ($\omega$) between the optical axes of the camera and the projector, which affects the geometrical and consequently mechanical design of the system. As $\omega$ is hidden in the rotation matrix ${R}^{p}$, it is rarely discussed in the FPP literature, to the best of our knowledge. Our target now is to delineate $\omega$ from ${R}^{p}$.

We mentioned earlier in Appendix A about CCS, which has been chosen as the world coordinate system. Similarly, there is a projector’s coordinate system (PCS). A point can be represented in both systems as ${\left[{x}^{c},{y}^{c},{z}^{c}\right]}^{T}$ and ${\left[{x}^{p},{y}^{p},{z}^{p}\right]}^{T}$, respectively. According to Eq. (A4) (see Supplementary Note 1), we have

18 $$\left[\begin{array}{c}{x}^{p}\\ {y}^{p}\\ {z}^{p}\end{array}\right]=\left[\begin{array}{ccc}{r}_{11}^{p} & {r}_{12}^{p} & {r}_{13}^{p}\\ {r}_{21}^{p} & {r}_{22}^{p} & {r}_{23}^{p}\\ {r}_{31}^{p} & {r}_{32}^{p} & {r}_{33}^{p}\end{array}\right]\left[\begin{array}{c}{x}^{c}\\ {y}^{c}\\ {z}^{c}\end{array}\right]+\left[\begin{array}{c}{t}_{1}^{p}\\ {t}_{2}^{p}\\ {t}_{3}^{p}\end{array}\right]$$

Let $\overrightarrow{{O}_{c}{A}_{c}}$ be the unit vector along the camera’s optical axis, we have ${O}_{c}^{c}={[0,0,0]}^{T}$ and ${A}_{c}^{c}={[\mathrm{0,0,1}]}^{T}$, expressed in CCS. By substituting them into Eq. (18), their coordinates in PCS can be obtained as ${O}_{c}^{p}={[{t}_{1}^{p},{t}_{2}^{p},{t}_{3}^{p}\,]}^{T}$ and ${{A}_{c}^{p}=\left[{r}_{13}^{p}+{t}_{1}^{p},{{r}_{23}^{p}+t}_{2}^{p},{r}_{33}^{p}+{t}_{3}^{p}\right]}^{T}$. Therefore, the directional vector of $\overrightarrow{{O}_{c}{A}_{c}}$ expressed in PCS is ${{\boldsymbol{e}}}_{{z}^{c}}^{p}={A}_{c}^{p}-{O}_{c}^{p}={[{r}_{13}^{p},{r}_{23}^{p},{r}_{33}^{p}]}^{T}$. Similarly, let $\overrightarrow{{O}_{c}{A}_{c}}$ be the unit vector along the projector’s optical axis, we have ${O}_{p}^{p}={[\mathrm{0,0,0}]}^{T}$ and ${A}_{p}^{p}={[\mathrm{0,0,1}]}^{T}$, expressed in PCS. Therefore, the directional vector of $\overrightarrow{{O}_{c}{A}_{c}}$ is ${{\boldsymbol{e}}}_{{z}^{p}}^{p}={A}_{p}^{p}-{O}_{p}^{p}={[\mathrm{0,0,1}]}^{T}$. Given ${{O}_{c}^{p}=\left[{t}_{1}^{p},{t}_{2}^{p},{t}_{3}^{p}\right]}^{T}$ and the projector’s axis ${{\boldsymbol{e}}}_{{z}^{p}}^{p}={\left[\mathrm{0,0,1}\right]}^{T}$, both in the PCS, we prove that when ${t}_{3}^{p}=0$, the ${{\boldsymbol{e}}}_{{z}^{p}}^{p}$ is perpendicular to the line connecting the optical centers of the projector and camera, i.e., the vector $\overrightarrow{{{O}_{p}^{p}O}_{c}^{p}}={\left[{t}_{1}^{p},{t}_{2}^{p},0\right]}^{T}$.

Now that both the directional vectors along the camera and the projector optical axes are represented in PCS, the angle between them can be readily computed as

19 $$\cos \left(\omega \right)={{\boldsymbol{e}}}_{{z}^{c}}^{p}\cdot {{\boldsymbol{e}}}_{{z}^{p}}^{p}={r}_{33}^{p}$$

We now return to the precision model for further simplification. First, as emphasized in our previous study, the first two terms of ${P}_{0}$ in Eq. (8), ${r}_{31}^{p}\frac{\left({u}^{c}-{u}_{0}^{c}\right)}{{f}_{u}^{c}}$ and ${r}_{32}^{p}\frac{\left({v}^{c}-{v}_{0}^{c}\right)}{{f}_{v}^{c}}$, varies spatially^22^. However, it is interesting to note that, in these two terms, (i) $\frac{{u}^{c}-{u}_{0}^{c}}{{f}_{u}^{c}}$ and $\frac{{v}^{c}-{v}_{0}^{c}}{{f}_{v}^{c}}$ represent the half FOV of the camera, which is less than 1 and further controllable by choosing a proper FOV; (ii) ${r}_{31}^{p}$ and ${r}_{33}^{p}$ are constrained by ${\left({r}_{31}^{p}\right)}^{2}+{\left({r}_{32}^{p}\right)}^{2}+{\left({r}_{33}^{p}\right)}^{2}=1$, which is also less than 1 and controllable by choosing a proper $\omega$, due to Eq. (19). As such, it is reasonable to omit these two terms (more detailed discussions are given in Part A of Discussions), so that Eq. (8) is simplified into:

20 $${P}_{0}={r}_{33}^{p}=\cos \left(\omega \right)$$

and subsequently, the IUPM in Eq. (16) is further derived into a simplified unified precision model (SUPM),

21 $${\sigma }_{{Z}_{M}}=\frac{\left|\cos \left(\omega \right)\right|{\left({z}^{w}\right)}^{2}}{{\left({B}_{e}\right)}_{M}}\frac{T}{2\pi }{\sigma }_{\varphi }$$

The merit of SUPM is its explicit geometrical expression, which can be used for practical FPP system design.

### B3: The simplified precision model variants towards system design

For the purpose of FPP system design, we consider a few SUPM variants for easy reference. First, we consider two cases of focal lengths: (I) ${f}_{u}^{p}{\boldsymbol{\ne }}{f}_{v}^{p}$ and (II) ${f}_{u}^{p}\approx {f}_{v}^{p}=f$, where case II is simpler yet highly possible. Accordingly, for case II, the SUPM in Eq. (21) is modified into

22 $${\sigma }_{{Z}_{M}}=\frac{\left|\cos \left(\omega \right)\right|{\left({z}^{w}\right)}^{2}}{{\left(B\right)}_{M}f}\frac{T}{2\pi }{\sigma }_{\varphi }$$

where

23 $${\left(B\right)}_{M}=\left\{\begin{array}{l}\begin{array}{cc}{t}_{1}^{p} & {for}\,M={Ver}3\end{array}\\ \begin{array}{cc}{t}_{2}^{p} & {for}\,M={Hor}3\end{array}\\ \begin{array}{cc}{B}_{0} & {for}\,M={OptE}3\end{array}\end{array}\right.$$

**Fig. 4:**
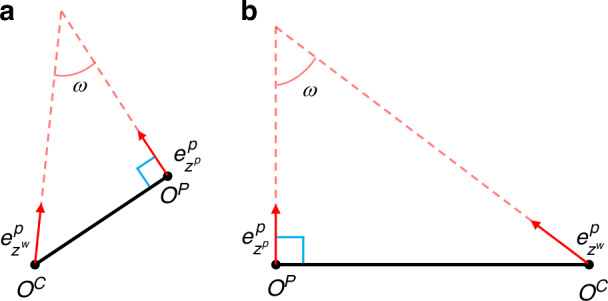
**FPP system design**. **a** Strategy I: the camera is fixed; (**b**) Strategy II: the projector is fixed

Next, we consider two strategies on parameter pre-determination and design. For Strategy I, ${z}^{w}$ is pre-determined by practical needs, and the projector positioning (thus ${B}_{e}$) and the camera direction (thus $\omega$) are designed according to precision requirement. This corresponds to the FPP configuration shown in Fig. 4a, where ${O}^{C}{O}^{P}$ remains perpendicular to the projector optical axes. For Strategy II, ${z}^{p}$ is pre-determined while the camera is adjustable, as shown in Fig. 4b. Under this scenario, we express ${z}^{w}={z}^{p}/\cos \left(\omega \right)$, and substitute it into the SUPM in Eq. (21). The original SUPM and the three variants are summarized in Table 1 for easy reference. In our current work, we consider $T$ shown in SUPM predefined by the phase-unwrapping algorithm^33^. It should be noted that Fig. 4 is only a simplified schematic. In practice, due to the measurement range of the optical system, ${z}^{p}$/ ${z}^{w}$ in Fig. 4 represents a range rather than a single value, and a more detailed analysis will be addressed in future work.

**Table 1:** Summary of FPP’ SUPM variants for design purpose (${{\boldsymbol{\sigma }}}_{{{\boldsymbol{Z}}}_{{\boldsymbol{M}}}}$)

	Case I: ${{\boldsymbol{f}}}_{{\boldsymbol{u}}}^{{\boldsymbol{p}}}\ne {{\boldsymbol{f}}}_{{\boldsymbol{v}}}^{{\boldsymbol{p}}}$		Case II: ${{\boldsymbol{f}}}_{{\boldsymbol{u}}}^{{\boldsymbol{p}}}\approx {{\boldsymbol{f}}}_{{\boldsymbol{v}}}^{{\boldsymbol{p}}}={\boldsymbol{f}}$	
	Precision Model	Designable variables	Precision Model	Designable variables
**Strategy I**: ${z}^{w}$ fixed	$\frac{\left|\cos \left(\omega \right)\right|{\left({z}^{w}\right)}^{2}}{{\left({B}_{e}\right)}_{M}}\frac{T}{2\pi }{\sigma }_{\varphi }$	$\left[{\left({B}_{e}\right)}_{M},\omega ,{\sigma }_{\varphi }\right]$ or $\left[{\left({B}_{e}\right)}_{M},\omega ,N,{\mu }_{e.{Sat}}\right]$	$\frac{\left|\cos \left(\omega \right)\right|{\left({z}^{w}\right)}^{2}}{{\left(B\right)}_{M}f}\frac{T}{2\pi }{\sigma }_{\varphi }$	$\left[{\left(B\right)}_{M},\omega ,{\sigma }_{\varphi }\right]$ or $\left[{\left(B\right)}_{M},\omega ,N,{\mu }_{e.{Sat}}\right]$
**Strategy II**: ${z}^{p}$ fixed	$\frac{1}{\left|\cos \left(\omega \right)\right|}\frac{{\left({z}^{p}\right)}^{2}}{{\left({B}_{e}\right)}_{M}}\frac{T}{2\pi }{\sigma }_{\varphi }$		$\frac{1}{\left|\cos \left(\omega \right)\right|}\frac{{\left({z}^{p}\right)}^{2}}{{\left(B\right)}_{M}f}\frac{T}{2\pi }{\sigma }_{\varphi }$	

To make the design even more practical, the phase error ${\sigma }_{\varphi }$ should also be specified, for which, detailed discussions have been provided in our previous work^22^. For initial system design, we adopt the following model^22^,

24 $${\sigma }_{\varphi }^{2}=\frac{4}{N{\mu }_{e.{Sat}}}$$

where *N* is the number of phase-shifting steps and ${\mu }_{e.{Sat}}$ is the saturation capacity of the sensor obtainable from sensor manufacturer. This model has the merit of simplicity, presents the lowest phase error, and serves as a precision limit. Apparently, higher *N* and ${\mu }_{e.{Sat}}$ improves precision, but the former takes longer data acquisition time while the latter indicates a higher-quality and usually more expensive camera. Thus, *N* and ${\mu }_{e.{Sat}}$ are considered as designable parameters, which is also reflected in Table 1. Based on Table 1, an FPP-Planner^34^ software is prototyped, with the details given in Part B of the Materials and Methods.

### C: Connection to other triangulation-based 3D reconstruction techniques

Since fringe projection, stereo vision and laser triangulation are all based on triangulation, they are naturally expected to have high similarity, or even considered as the same, in a “vague” manner. For example, since Zhang insightfully regarded the projector as an inverse camera in 2006^18^, FPP has been intuitively considered as stereo vision, which is known to many researchers and practitioners. In addition, such similarity can also be appreciated from the viewpoint of the principle of Helmholtz reciprocity, where the projector in FPP acts as a virtual camera for each pixel of the real camera, as has already been widely recognized in the single-pixel image^35–37^. With our precision models established in this paper, we are now able to confirm their equivalence quantitatively, anchoring at precision which is a major concern in metrology. Such treatment also extends to laser triangulation. Since each triangulation-based method has its own optical, mechanical and algorithmic characteristics, such connection does not mean that our FPP precision models can be immediately used to guide the design of other triangulation-based methods.

### C1: Stereo vision

A typical stereo vision system consists of two cameras. Unlike FPP, it does not actively project light onto the object but passively captures images. As a result, the correspondence process in stereo systems is generally more challenging than in FPP. A common approach combines epipolar constraints with intensity-based correspondence. To simplify this process, the most widely used strategy is epipolar rectification, which aligns corresponding points along the same horizontal image lines^38^. After rectification, the rotation matrix *R* becomes identity matrix, and the translation vector **t** typically satisfies ${t}_{2}={t}_{3}=0$. The coordinate system of the left camera is chosen as WCS. The conventional precision model for stereo vision systems is^3^,

25 $${\sigma }_{z}=\frac{{\left({z}^{w}\right)}^{2}}{{B}_{0}f}{\sigma }_{d}$$

where ${B}_{0}$ is the traditional baseline; *f* is the camera focal length; ${\sigma }_{d}$ is the precision of pixel correspondence in spatial distance. Note that, the original form in^3^ uses *δ*_*z*_ and *δ*_*d*_ in Eq. (25), both of which are random variables. We have used their standard deviations instead.

We further examine the parameters of SUPM in Eq. (21). First, we consider a macroscopic system, where $\omega$ is small, say, $\omega \le {10}^{^\circ }$, and $\cos \left(\omega \right)\approx 1$; second, we look at a normal case that ${f}_{u}^{p}={f}_{v}^{p}=f$, so that ${\left({B}_{e}\right)}_{M}={\left(B\right)}_{M}f$; third, we convert the pixel correspondence in phase to that in spatial distance, and re-write $T/2\pi \times {\sigma }_{\varphi }={\sigma }_{d}$. As a result, our FPP’s SUPM model becomes

26 $${\sigma }_{{Z}_{M}}=\frac{{\left({z}^{w}\right)}^{2}}{{\left(B\right)}_{M}f}{\sigma }_{d}$$

The only explicit difference between Eqs. (25) and (26) is ${\left({B}_{e}\right)}_{M}$. Note that for the OptE3 method, i.e., $M={OptE}3$, we have ${\left(B\right)}_{M}={B}_{0}$, making Eqs. (25) and (26) completely identical. This fact not only equivales FPP and stereo vision with respect to (w.r.t) precision but also highlights the theoretical significance of the OptE3 method.

### C2: Laser triangulation

As mentioned in Introduction, the source of noise in laser triangulation is speckle. Regarding the impact of speckle noise on the precision limit of laser triangulation, the Hausler research group conducted in-depth studies and determined the ultimate limit of uncertainty to be^2,23^,

27 $${\delta }_{z}=\frac{C}{2\pi }\frac{\lambda }{\sin ({u}_{{obs}})\sin ({\rm{\omega }})}$$

where C is the speckle contrast, $\lambda$ is the laser wavelength, ${u}_{{obs}}$ is the observation aperture. In refs. ^2,23^, the angle is denoted as $\theta$; however, to maintain consistency with this paper, we replace it with ${\rm{\omega }}$. Based on geometrical optics and Fig. 1 of ref. ^23^, $\sin ({u}_{{obs}})$ can be approximated as:

28 $$\sin \left({u}_{{obs}}\right)=\frac{D}{2{z}^{w}}$$

where *D* is the diameter of the camera’s aperture diaphragm. By substituting Eq. (28) into Eq. (27), we have,

29 $${\delta }_{z}=\frac{C\lambda }{\pi D}\frac{{z}^{w}}{\sin ({\rm{\omega }})}\propto \frac{{z}^{w}}{\sin \left({\rm{\omega }}\right)}$$

By assuming that *D* is treated as a constant, Eq. (29) highlights the influence of geometry.

For FPP, we consider OptE3 only and assume that ${f}_{u}^{p}={f}_{v}^{p}=f$, so that ${B}_{e0}={B}_{0}f={z}^{w}f\sin \left(\omega \right)$. as shown in Fig. 4. Consequently, Eq. (21) can be rewritten as,

30 $${\sigma }_{{Z}_{M}}=\frac{{z}^{w}\cos \left(\omega \right)}{\sin ({\rm{\omega }})}\frac{T{\sigma }_{\varphi }}{2\pi f}$$

When $\omega$ is small, say, $\omega \le {10}^{^\circ }$, which is usually the case in a laser triangulation system, we have $\cos \left(\omega \right)\ge \cos \left(10{\rm{^\circ }}\right)=0.985$. We thus apply ${\rm{c}}{\rm{os}}\left({\rm{\omega }}\right)\approx 1$ onto Eq. (30), yielding,

31 $${\sigma }_{{Z}_{M}}\approx \frac{{z}^{w}}{\sin ({\rm{\omega }})}\frac{T{\sigma }_{\varphi }}{2\pi f}\propto \frac{{z}^{w}}{\sin \left({\rm{\omega }}\right)}$$

By comparing the two models presented in Eqs. (29) and (31), it is evident that the precisions of laser triangulation and FPP are indeed consistent w.r.t system geometry.

As this paper involves a number of precision models, they are summarized in Table 2 for convenience.

**Table 2:** Summary of unified precision models in this paper

Models	Equations	Properties
GUPM [Eq. (1)]	${\sigma }_{{z}_{M}}=\frac{P}{{\alpha }_{M}}\frac{T}{2\pi }{{\rm{\sigma }}}_{\varphi }$	(i) Feature: general and fundamental; (ii) Finding: the general relationship with a precision right-angle triangle (PRT); (iii) Application: for precision evaluation after calibration (post-evaluation).
IUPM [Eq. (16)]	${\sigma }_{{Z}_{M}}=\frac{\left|{P}_{0}\right|{\left({z}^{w}\right)}^{2}}{{\left({B}_{e}\right)}_{M}}\frac{T}{2\pi }{\sigma }_{\varphi }$	(i) Feature: Incorporates a special yet not restrictive configuration (${t}_{3}^{p}=0$); (ii) Findings: the special relationship with an effective baseline right-angle triangle (EBRT), and the influence of the baselines on the measurement precision; (iii) Application: As an intermediate bridge between GUPM and SUPM.
SUPM [Eq. (21)]	${\sigma }_{{Z}_{M}}=\frac{\left|\cos \left(\omega \right)\right|{\left({z}^{w}\right)}^{2}}{{\left({B}_{e}\right)}_{M}}\frac{T}{2\pi }{\sigma }_{\varphi }$	(i) Feature: physics-intuitive and practical for use (ii) Finding: the influence of the optical axis angle $\omega$ on the measurement precision; (iii) Application: Guides tolerance allocation in system design (pre-evaluation).
Reformulated from SUPM [Eq. (26)]	${\sigma }_{{Z}_{M}}=\frac{{\left({z}^{w}\right)}^{2}}{{\left({B}_{e}\right)}_{M}}{\sigma }_{d}$	(i) Links to stereo vision.
Reformulated from SUPM [Eq. (31)]	${\sigma }_{{Z}_{M}}\propto \frac{{z}^{w}}{\sin \left({\rm{\omega }}\right)}$	(i) Links to laser triangulation.

## Discussions

Theoretical understanding of the measurement precision of FPP is important in practical metrology. It is thus important to discuss the possible gaps between theory and practice.

### A: The assumptions in model establishment

#### From GUPM to IUPM

We assume that ${t}_{3}^{p}=0$. In fact, it is difficult to satisfy strictly. We check, to what extent, this assumption remains reasonable. By using Eq. (A11), we calculate the derivatives of ${\theta }_{{opt}}$ w.r.t ${t}_{1}^{p}$ and ${t}_{2}^{p}$. Fortunately, we find that even when ${t}_{3}^{p}\ne 0$, Eqs. (8–17) can still be valid, as long as the ratio ${R}_{t}$ is sufficiently large, with ${R}_{t}$ defined as,

32 $${R}_{t}=\max \left({t}_{1}^{p}/{t}_{3}^{p},{t}_{2}^{p}/{t}_{3}^{p}\right)$$

where max(.,.) takes the larger value of the two input arguments. A similar perspective, where the approximation holds when ${t}_{3}^{p}$ has a very small value compared with ${t}_{1}^{p}$ or ${t}_{2}^{p}$ has also been proposed in ref. ^39^. We conducted simulations and real experiments with fifteen different configurations of FPP systems, and found that, with the condition of ${R}_{t}\ge 30$, the relative errors of the optimal angles obtained from Eqs. (A11) and (A12) do not exceed 0.7%.

#### From IUPM to SUPM

We neglect the first two terms on the right-hand side of Eq. (8) through analysis ((see Supplementary Note 3 for details for details). With the two notes in that analysis, and without loss of generality, we further consider the case where the horizontal and vertical FOVs are identical, then we can readily estimate the values of the first two terms relative to the third term in Eq. (8). For example, by reasonably taking ${\rm{FOV}}={20}^{\circ }$ and $\omega \le 10{\rm{^\circ }}$ ^21,32^, we find that the sum of the first two terms is no more than 4.4% of the third term. In practice, as shown in our extensive experiments, this approximation error is typically below 2%, where the horizontal and vertical half-FOV angles are about 9.64° and 8.01°, and the range of $\omega$ is about from 4.5° to 8°.

### B: Applicability of unified precision models

Since the GUPM is established under the pinhole imaging assumption, it is applicable to FPP systems as long as this assumption holds and the calibration quality is satisfactory. The simplified models, IUPM and SUPM, are derived from GUPM through controlled approximations and remain valid when the associated approximation errors are tolerable, with the advantages of clearer physical interpretation and more efficient system design.

### C: Model sensitivity to error sources (FPP system variables)

We also analyze the model sensitivity to error sources. As the GUPM involves a large number of system parameters, we focus on the SUPM. The SUPM presented by Eq. (21) is related to system variables of $\omega$, ${z}^{w}$, ${\left({B}_{e}\right)}_{M}$ and ${\sigma }_{\varphi }$. The sensitivity of ${\sigma }_{{Z}_{M}}$ w.r.t. these variables reflect the gap between design and configuration. We define the relative error of a variable *f* as ${\epsilon }_{f}={\delta }f/f$ and the sensitivity of *f* w.r.t. an error source *g* as $\partial f/\partial g$, we then obtain,

33 $${\epsilon }_{{\sigma }_{{Z}_{M}}}(\omega )={s}_{g}{\epsilon }_{g}$$

where ${s}_{\omega }=\tan \left(\omega \right)\omega$ (≈ 0.03 when $\omega =10$°); ${s}_{{z}^{w}}=2$; ${s}_{{\left({B}_{e}\right)}_{M}}=1$; ${s}_{{\sigma }_{\varphi }}=1$, thus the error source of $\omega$ can be ignored and the measurement precision is most sensitive to the measurement distance. As a trial result to understand the system precision, we can assume that the relative errors are the same, i.e, ${\epsilon }_{g}=\epsilon$, then the total relative error of the system precision can be expressed as,

34 $${\epsilon }_{{\sigma }_{{Z}_{M}}}={\left[{\left({s}_{{z}^{w}}\right)}^{2}+{\left({s}_{{\left({B}_{e}\right)}_{M}}\right)}^{2}+{\left({s}_{{\sigma }_{\varphi }}\right)}^{2}\right]}^{1/2}\epsilon \approx \sqrt{6}\epsilon$$

which means that the relative error in the system parameters could be magnified, in the worst case, by $\sqrt{6}\approx 2.45$ times. For example, if these system variables have been deviated by 10%, it will result in 24.5% deviation in the system precision. Such a magnification can be easily remedied by setting a more stringent target precision. As will be seen in Part B5 of Materials and Methods, when a reconstruction precision of ${\sigma }_{{z\_TP}}$ is required, a more stringent precision is set at ${\sigma }_{{z\_TP}}/2$.

### D: Influence of less collaborative object shapes

The proposed models are independent of the global object geometry, since FPP is a point-wise measurement technique. For object surfaces with edges and high slopes, reduced fringe reflectance or partial occlusion mainly manifests as changes in fringe contrast, which are accommodated by the phase-noise model ${M}_{P-F}$ ^22^ adopted in the unified models. For objects with multi-material surfaces—an important and actively studied topic^40,41^ —the projected fringes may be affected by inter-reflection or subsurface scattering, causing the phase variance term to deviate from the strict form assumed in ${M}_{P-F}$. Nevertheless, the unified precision model proposed in this work can still serve as a useful first-order approximation for analyzing precision trends.

### E: Influence of other possible factors

There are other factors that could affect the FPP measurement precision:

(i) Give an FPP setup, the calibration uncertainty of system parameters not only affects measurement accuracy, but also measurement precision as these parameters present in our precision models;
(ii) Projector nonlinearity is one of the well-noticed error sources, but it is not explicitly considered in this paper, as effective correction methods are well established and such effects are no longer a dominant limitation in modern projection systems^19,42^.
(iii) Practical environment may deviate the measurement result from its theorical prediction, including the surface texture properties of the test sample, vibrations and ambitious lighting.

While they are worth studying, our considerations in this paper are as follows:

(i) In real practice, it is encouraged that the measurement environment should be maintained towards ideal, if possible;
(ii) With an non-ideal environment, as discussed in Part B of this section, we suggest to adopt a more stringent target precision during the system design phase. Then, even when the environment is harsh, our theoretical models can still output precision limit and thus serve as a guideline of measurement capability and possible improvement.
(iii) In our extensive experimental validations in a conventional laboratory, presented in Materials and Methods, the theoretical and experimental results match satisfactorily, even with the influence of various factors. As an example, our experimental results show that the discrepancy between theoretical predictions and measured precision remains within 5%^22^. As another example, our theoretical model-based FPP-planner successfully guides the practical system design in Part B5 of Materials and Methods of this paper.

## Materials and methods

FPP has been studied for more than four decades, but there is a lack of sufficiently deep discussion on theoretical precision models. We thus adopt a theoretical approach to enhance the understanding of the FPP techniques. Despite their complexity in formulation, we formally derive theoretical expressions of Ver3/Hor3/OptE3, and put them together, for the first time. This enables us to see the tight connections from their complicated appearances. These formulations are given in Part A below, while our findings from them have been described in Results and Discussions earlier. Furthermore, all the findings are supported by our comprehensive validations, which are given in Part B of this section.

### A. Precision models of Ver3, Hor3 and OptE3

For Ver3, we denote the absolute phase distribution obtained in the image coordinate system in Step (ii) in Supplementary Note 2 as ${\varphi }^{{Ver}3}\left({u}^{c},{v}^{c}\right)$, where we use $\left({u}^{c},{v}^{c}\right)$ and $\left({u}^{p},{v}^{p}\right)$ to indicate a camera pixel and a project pixel, respectively. The phase of the projected vertical fringe is designed to increase horizontally and linearly. Then the correspondence process turns out to be^22^,

35 $${u}^{p}=\frac{{\varphi }^{{Ver}3}\left({u}^{c},{v}^{c}\right)}{2\pi }T$$

where $T$ is the fringe period. In the reconstruction process, we substitute Eq. (A2) and (A3) into Eq. (A5) see Supplementary Note 1 and obtain,

36 $${z}_{{Ver}3}^{w}\left({u}^{p}\right)=\frac{{t}_{3}^{p}\left({u}^{p}-{u}_{0}^{p}\right)-{f}_{u}^{p}{t}_{1}^{p}}{\left\{\begin{array}{c}\left[{f}_{u}^{p}{r}_{13}^{p}-{r}_{33}^{p}\left({u}^{p}-{u}_{0}^{p}\right)\right]+\frac{{u}^{c}-{u}_{0}^{c}}{{f}_{u}^{c}}\left[{{f}_{u}^{p}r}_{11}^{p}-{r}_{31}^{p}\left({u}^{p}-{u}_{0}^{p}\right)\right]\\ +\frac{{v}^{c}-{v}_{0}^{c}}{{f}_{v}^{c}}\left[{f}_{u}^{p}{r}_{12}^{p}-{r}_{32}^{p}\left({u}^{p}-{u}_{0}^{p}\right)\right]\end{array}\right\}}$$

where the reconstructed depth is represented by pixel coordinates and intrinsic and extrinsic parameters of the camera and the projector. Then, according to Eq. (35), we have ${\sigma }_{{u}^{p}}^{2}={\left(\frac{T}{2\pi }\right)}^{2}{\sigma }_{{\varphi }^{{Ver}3}}^{2}$ where $\sigma$ represents standard deviation; further according to Eq. (35), ${u}^{p}$ is the only variable for a calibrated FPP system, and thus we have ${\sigma }_{{z}_{{Ver}3}}^{2}={\left(\frac{\partial {z}_{{Ver}3}^{w}}{\partial {u}^{p}}\right)}^{2}{\sigma }_{{u}^{p}}^{2}$. By combining these two results, the variance of the measured depth can be derived as,

37 $${\sigma }_{{z}_{{Ver}3}}^{2}=\frac{{\left(\frac{T}{2\pi }\right)}^{2}{\left[\begin{array}{c}{r}_{31}^{p}\frac{\left({u}^{c}-{u}_{0}^{c}\right)}{{f}_{u}^{c}}{z}^{w}+{r}_{32}^{p}\frac{\left({v}^{c}-{v}_{0}^{c}\right)}{{f}_{v}^{c}}{z}^{w}\\ +{r}_{33}^{p}{z}^{w}+{t}_{3}^{p}\end{array}\right]}^{4}}{{\left\{{f}_{u}^{p}\left[\begin{array}{c}\left({r}_{11}^{p}{t}_{3}^{p}-{t}_{1}^{p}{r}_{31}^{p}\right)\frac{\left({u}^{c}-{u}_{0}^{c}\right)}{{f}_{u}^{c}}+\\ \left({r}_{12}^{p}{t}_{3}^{p}-{t}_{1}^{p}{r}_{32}^{p}\right)\frac{\left({v}^{c}-{v}_{0}^{c}\right)}{{f}_{v}^{c}}+\left({r}_{13}^{p}{t}_{3}^{p}-{t}_{1}^{p}{r}_{33}^{p}\right)\end{array}\right]\right\}}^{2}}{\sigma }_{{\varphi }^{{Ver}3}}^{2}$$

which was obtained earlier in^22^.

For Hor3, the derivation of the depth variance is similar to that of Ver3, except that (a) horizontal fringes are used for projection, (b) ${u}^{p}$ is replaced by ${v}^{p}$ in Eq. (1) for correspondence, and (c) Eqs. (A2), (A3) and (A6) (see Supplementary Note 1)are used to reconstruct ${z}_{H{or}3}^{w}$. The precision model is derived as,

38 $${\sigma }_{{z}_{Hor3}}^{2}=\frac{{\left(\frac{T}{2\pi }\right)}^{2}{\left[\begin{array}{c}{r}_{31}^{p}\frac{\left({u}^{c}-{u}_{0}^{c}\right)}{{f}_{u}^{c}}{z}^{w}+{r}_{32}^{p}\frac{\left({v}^{c}-{v}_{0}^{c}\right)}{{f}_{v}^{c}}{z}^{w}\\ +{r}_{33}^{p}{z}^{w}+{t}_{3}^{p}\end{array}\right]}^{4}}{{\left\{{f}_{v}^{p}\left[\begin{array}{c}\left({r}_{21}^{p}{t}_{3}^{p}-{t}_{2}^{p}{r}_{31}^{p}\right)\frac{\left({u}^{c}-{u}_{0}^{c}\right)}{{f}_{u}^{c}}+\\ \left({r}_{22}^{p}{t}_{3}^{p}-{t}_{2}^{p}{r}_{32}^{p}\right)\frac{\left({v}^{c}-{v}_{0}^{c}\right)}{{f}_{v}^{c}}+\left({r}_{23}^{p}{t}_{3}^{p}-{t}_{2}^{p}{r}_{33}^{p}\right)\end{array}\right]\right\}}^{2}}{\sigma }_{{\varphi }^{{Hor}3}}^{2}$$

For OptE3, oriented fringe patterns are projected with the phase distribution designed as^20^

39 $${\varphi }^{{OptE}3}\left({u}^{c},{v}^{c}\right)=\frac{2\pi }{T}\left[{u}^{p}sin\left({\theta }_{{opt}}\right)+{v}^{p}cos\left({\theta }_{{opt}}\right)\right]$$

By solving Eqs. (A7) (see Supplementary Note 1) and (39), the correspondence result is,

40 $${u}^{p}=\frac{{l}_{2}{\varphi }^{{OptE}3}\left({u}^{c},{v}^{c}\right)+\frac{2\pi }{T}{l}_{3}cos\left({\theta }_{{opt}}\right)}{\frac{2\pi }{T}\left[{l}_{2}sin\left({\theta }_{{opt}}\right)-{l}_{1}cos\left({\theta }_{{opt}}\right)\right]}$$

41 $${v}^{p}=\frac{{l}_{1}{\varphi }^{{OptE}3}\left({u}^{c},{v}^{c}\right)+{\frac{2\pi }{T}l}_{3}sin\left({\theta }_{{opt}}\right)}{\frac{2\pi }{T}\left[{l}_{1}cos\left({\theta }_{{opt}}\right)-{l}_{2}sin\left({\theta }_{{opt}}\right)\right]}$$

Recall that in OptE3, only three equations are needed for reconstruction. Thus, we can either follow the derivation for Ver3 using Eqs. (A2), (A3) and (A5) (see Supplementary Note 1), with ${u}^{p}$ given in Eq. (40), or follow the derivation for Hor3 using Eqs. (A2), (A3) and (A6), with ${v}^{p}$ given in Eq. (41). While both approaches yield the same result, we select the former for explanation. The following result can be derived, which was first obtained in^20^,

42 $${\sigma }_{{z}_{{OptE}3}}^{2}\left({u}^{p}\right)=\frac{{\left(\frac{T}{2\pi }\right)}^{2}{\left[\begin{array}{c}{r}_{31}^{p}\frac{\left({u}^{c}-{u}_{0}^{c}\right)}{{f}_{u}^{c}}{z}^{w}+{r}_{32}^{p}\frac{\left({v}^{c}-{v}_{0}^{c}\right)}{{f}_{v}^{c}}{z}^{w}\\ +{r}_{33}^{p}{z}^{w}+{t}_{3}^{p}\end{array}\right]}^{4}}{{\left\{{f}_{u}^{p}\left[\begin{array}{c}\left({r}_{11}^{p}{t}_{3}^{p}-{t}_{1}^{p}{r}_{31}^{p}\right)\frac{\left({u}^{c}-{u}_{0}^{c}\right)}{{f}_{u}^{c}}+\\ \left({r}_{12}^{p}{t}_{3}^{p}-{t}_{1}^{p}{r}_{32}^{p}\right)\frac{\left({v}^{c}-{v}_{0}^{c}\right)}{{f}_{v}^{c}}+\left({r}_{13}^{p}{t}_{3}^{p}-{t}_{1}^{p}{r}_{33}^{p}\right)\end{array}\right]\right\}}^{2}}\frac{{\left({l}_{2}\right)}^{2}}{{\left({l}_{1}\right)}^{2}+{\left({l}_{2}\right)}^{2}}{\sigma }_{{\varphi }^{{OptE}3}}^{2}$$

Here, we move one step further by substituting Eqs. (A8) and (A9) (see Supplementary Note 1) into Eq. (42), and obtain, for the first time, the following precision model for OptE3,

43 $${\sigma }_{{z}_{{OptE}3}}^{2}=\frac{{\left(\frac{T}{2\pi }\right)}^{2}{\left[\begin{array}{c}{r}_{31}^{p}\frac{\left({u}^{c}-{u}_{0}^{c}\right)}{{f}_{u}^{c}}{z}^{w}+{r}_{32}^{p}\frac{\left({v}^{c}-{v}_{0}^{c}\right)}{{f}_{v}^{c}}{z}^{w}+\\ {r}_{33}^{p}{z}^{w}+{t}_{3}^{p}\end{array}\right]}^{4}}{\left(\begin{array}{c}{\left\{{f}_{u}^{p}\left[\begin{array}{c}\left({r}_{11}^{p}{t}_{3}^{p}-{t}_{1}^{p}{r}_{31}^{p}\right)\frac{\left({u}^{c}-{u}_{0}^{c}\right)}{{f}_{u}^{c}}+\\ \left({r}_{12}^{p}{t}_{3}^{p}-{t}_{1}^{p}{r}_{32}^{p}\right)\frac{\left({v}^{c}-{v}_{0}^{c}\right)}{{f}_{v}^{c}}+\left({r}_{13}^{p}{t}_{3}^{p}-{t}_{1}^{p}{r}_{33}^{p}\right)\end{array}\right]\right\}}^{2}\\ +{\left\{{f}_{v}^{p}\left[\begin{array}{c}\left({r}_{21}^{p}{t}_{3}^{p}-{t}_{2}^{p}{r}_{31}^{p}\right)\frac{\left({u}^{c}-{u}_{0}^{c}\right)}{{f}_{u}^{c}}+\\ \left({r}_{22}^{p}{t}_{3}^{p}-{t}_{2}^{p}{r}_{32}^{p}\right)\frac{\left({v}^{c}-{v}_{0}^{c}\right)}{{f}_{v}^{c}}+\left({r}_{23}^{p}{t}_{3}^{p}-{t}_{2}^{p}{r}_{33}^{p}\right)\end{array}\right]\right\}}^{2}\end{array}\right)}{\sigma }_{{\varphi }^{{OptE}3}}^{2}$$

It is worth clarifying that, the Ver3 precision model in Eq. (37) has already been obtained in^22^; the Hor3 precision model in Eq. (38) is trivial by simply mimicking the derivation for Ver3; the OptE3 precision model has already been partially obtained in the form of Eq. (42) in^20^. We only add a tiny and trivial step to change the model into a new form in Eq. (43), which was not obtained or favored earlier as it looks more complicated. However, these seemingly trivial works enable us to see the important and surprising similarity among these models, leading to our work shown in Results and Discussions.

### B. Experimental validations

In order to validate our theoretical analysis results, we developed a series of FPP systems with different configurations using a DLP Projector (Model: DLP6500, Resolution:1080×1920) and a camera (Model: MER2-502-79U3M-L) equipped with a 25 mm lens (Model: HN-P-2524-20M). All the systems satisfy ${R}_{t}\ge 30$, as discussed in Eq. (32). Zhang and Huang’s method is used to calibrate the systems^18^. The three-step phase-shifting method^43^ and the optimum three-frequency method^44^ are adopted for wrapped phase retrieval and absolute phase recovery, respectively. Other advanced phase-unwrapping algorithms^45–47^ can also be an option.

### B1. Validation of the constant optimal angle

**Fig. 5:**
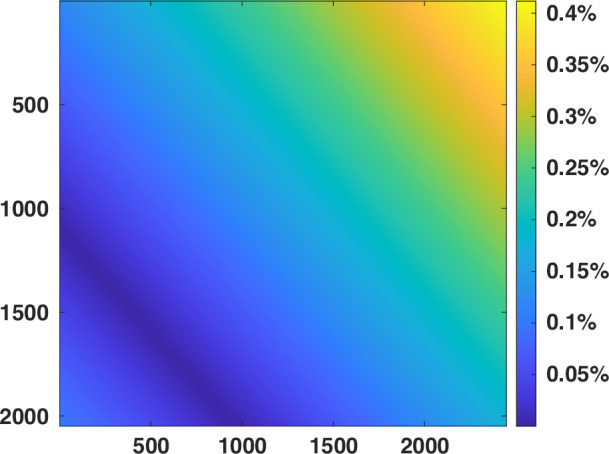
The relative errors of ${\theta }_{{opt}}$ calculated by Eqs. (A12) and (12) for FPP #1

Since ${\theta }_{{opt}}$ appears in both general and special Pythagoras relationships as shown in Fig. 3 and thus plays an essential role in an FPP system, we first validate that it is nearly constant, as given in Eq. (12). We construct ten FPP systems (FPP #1-#10) with different camera and projector locations. In these systems, the horizontal projection baseline is greater than the vertical projection baseline (${B}_{e1} > {B}_{e2}$), resulting in ${\pi /4\le \theta }_{{opt}}\le 3\pi /4$. As an example, for the first FPP system (FPP #1), the intrinsic and extrinsic matrices are obtained through calibration as,

44 $${A}^{c}=\left[\begin{array}{ccc}7323.853 & 0 & 1214.116\\ 1 & 7321.837 & 1012.628\\ 0 & 0 & 1\end{array}\right]$$

45 $${A}^{p}=\left[\begin{array}{ccc}3397.550 & 0 & 913.608\\ 1 & 3402.537 & 493.024\\ 0 & 0 & 1\end{array}\right]$$

46 $$\left[\begin{array}{cc}{R}^{p} & {t}^{p}\end{array}\right]=\left[\begin{array}{cccc}0.975 & 0.017 & 0.220 & -85.924\\ -0.026 & 0.999 & 0.041 & -83.360\\ -0.220 & -0.046 & 0.975 & 1.105\end{array}\right]$$

with which, ${\theta }_{{opt}}$ can be calculated by Eq. (A11) with the value fluctuating with $\left({u}^{c},{v}^{c}\right)$, and also calculated by Eq. (12) to be ${\theta }_{{opt}}=0.7998$. Their difference is shown in Fig. 5, where the maximum relative error is lower than 0.5%.

### B2. Validation of the Pythagoras relationships

We now move on to validate the Pythagorean relationships, PRT and EBRT. Interestingly, the EBRT relationship in Fig. 3b, which is geometrical, is naturally true. We thus turn to validate PRT in Eqs. (6) and (7), where the precision of three methods, Ver3/Hor3/OptE3, are separately measured. By defining

47 $$Q={\left[{\left({\sigma }_{{OptE}3}/{\sigma }_{{Ver}3}\right)}^{2}+{\left({\sigma }_{{OptE}3}/{\sigma }_{{Hor}3}\right)}^{2}\right]}^{\frac{1}{2}}$$

then both Eqs. (6) and Eq. (7) are equivalent to $Q=1$, which will be validated.

A ceramic plate with a peak-valley difference less than 0.005 mm is measured by all the three methods. To reduce the effect of the unavoidable distortion from the lenses, the central 400×480 pixels of the camera are used as a region of interest (ROI). The point cloud data (${x}_{0}^{w},{y}_{0}^{w},{z}_{0}^{w}$) in each ROI are fitted into a plane as

48 $${A}_{1}{x}^{w}+{B}_{1}{y}^{w}+{C}_{1}{z}^{w}+{D}_{1}=0$$

where the plane parameters ${A}_{1}$, ${B}_{1}$, ${C}_{1}$ and ${D}_{1}$ are obtained by the fitting. For each measured object point $\left({x}_{0}^{w},{y}_{0}^{w},{z}_{0}^{w}\right)$, its closest distance to the fitted plane is calculated by

49 $$d\left({x}_{0}^{w},{y}_{0}^{w},{z}_{0}^{w}\right)=\frac{{A}_{1}{x}_{0}^{w}+{B}_{1}{y}_{0}^{w}+{C}_{1}{z}_{0}^{w}+{D}_{1}}{\sqrt{{A}_{1}^{2}+{B}_{1}^{2}+{C}_{1}^{2}}}$$

from which, the standard deviation (STD) is calculated from the ROI as the experimental precision.

Table 3 shows the measurement results for FPP #1. Using this system, the plate is placed at ten different positions and then measured, which is indicated by Column 1. For each position, the precisions by the three different methods are calculated and shown in Columns 2-4, respectively; ${\sigma }_{{OptE}3}/{\sigma }_{{Ver}3}$ and ${\sigma }_{{OptE}3}/{\sigma }_{{Hor}3}$ are subsequently calculated and shown in Columns 5-6, respectively; the Q value is calculated in Column 7. It can be observed that the measurement results for ten different places are close to one another. To ease the interpretation and illustration below, we also calculate the mean values of Columns 5-7, as shown in the last row of Table 3. It can be seen that the mean value of Q is 0.992, which is very close to the ideal value of 1, with a relative error of merely 0.8%. Meanwhile, the mean value of Columns 5 and 6 are 0.705 and 0.696, respectively, which are very close to the theoretical results of 0.717 and 0.697 calculated using Eq. (6), with relative errors of 1.6% and 0.12%, respectively.

**Table 3:** Measurement precision of the standard plane for FPP #1 (${{\boldsymbol{\theta }}}_{{\boldsymbol{opt}}}$ = 0.801 rad)

Positions	${\sigma }_{{Ver}3}$/mm	${\sigma }_{{Hor}3}$/mm	${\sigma }_{{OptE}3}$/mm	$\frac{{\sigma }_{{OptE}3}}{{\sigma }_{{Ver}3}}$	$\frac{{\sigma }_{{OptE}3}}{{\sigma }_{{Hor}3}}$	*Q*
1	0.128	0.130	0.090	0.703	0.692	0.986
2	0.124	0.127	0.088	0.709	0.693	0.992
3	0.120	0.121	0.085	0.708	0.703	0.998
4	0.133	0.135	0.093	0.699	0.689	0.981
5	0.126	0.128	0.088	0.698	0.688	0.980
6	0.111	0.113	0.079	0.712	0.699	0.998
7	0.119	0.120	0.085	0.714	0.708	1.006
8	0.125	0.127	0.089	0.712	0.701	0.999
9	0.130	0.131	0.091	0.700	0.695	0.986
10	0.136	0.138	0.096	0.706	0.696	0.992
Mean	-	-	-	0.706	0.696	0.992
Theoretical	-	-	-	0.717	0.697	1
Relative error	-	-	-	1.6%	0.12%	0.8%

**Fig. 6:**
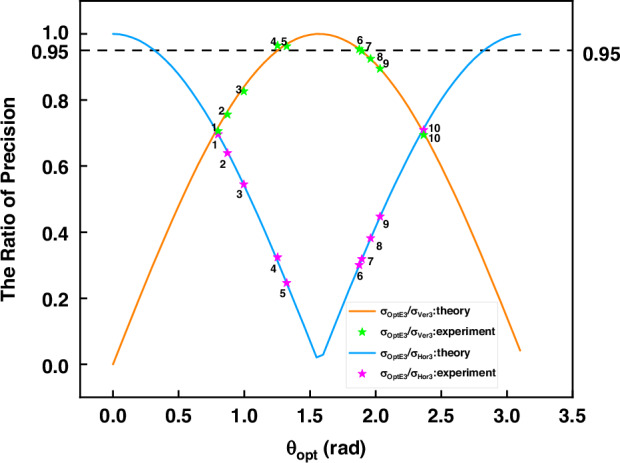
Ratio of precision measured between Ver3(Hor3) with OptE3 in different FPP systems (#1-#10)

Based on FPP #1, we gradually move the camera horizontally away from the projector, and ${\theta }_{{opt}}$ changes accordingly from $\pi /4$ to $\pi /2$ (FPP #2-#5). Furthermore, we place the camera on the other side of the projector so that ${\theta }_{{opt}}$ changes from $\pi /2$ to $3\pi /4$ (FPP #6-#10). For each system, we repeat the above verification. In Fig. 6, the mean values of ${\sigma }_{{OptE}3}/{\sigma }_{{Ver}3}$ and ${\sigma }_{{OptE}3}/{\sigma }_{{Hor}3}$ measured in each FPP system are shown with green and magenta stars, respectively, while the theoretical values are plotted as the orange and blue lines for comparison. The perfect agreement has been achieved for all the ten systems.

**Fig. 7:**
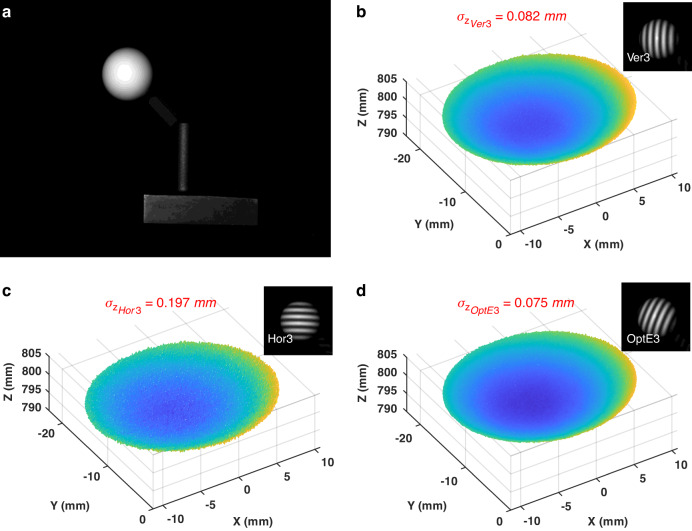
Experimental results of the standard sphere. **a** the standard sphere; **b**–**d** the measurement point clouds by Ver3/Hor3/OptE3 methods in system (FPP #11)

To further validate our analysis, we also repeatedly measured a standard sphere with a diameter of 30.0105 mm and a sphericity of 0.002 mm. For this purpose, we built an FPP#11 system, whose optimal angle is ${\theta }_{{Opt}}=68.096^\circ$. Figure 7a shows a photo of the standard sphere. Figure 7b–d presents the corresponding measured point clouds by Ver3/Hor3/OptE3, respectively. For each measured point $\left({x}_{0}^{w},{y}_{0}^{w},{z}_{0}^{w}\right)$, we performed a sphere fitting and then computed the closest distance from each point to the fitted sphere. The standard deviation of these closest distances was taken as the experimental precision. Substituting this precision into Eq. (47) yields $Q=0.9907$, (corresponds to a relative error of 0.93%), against the theoretical value $Q=1$. This result shows that the measurement precision of the sphere is consistent with that of the plane.

### B3. Validation of the influence of baselines

**Fig. 8:**
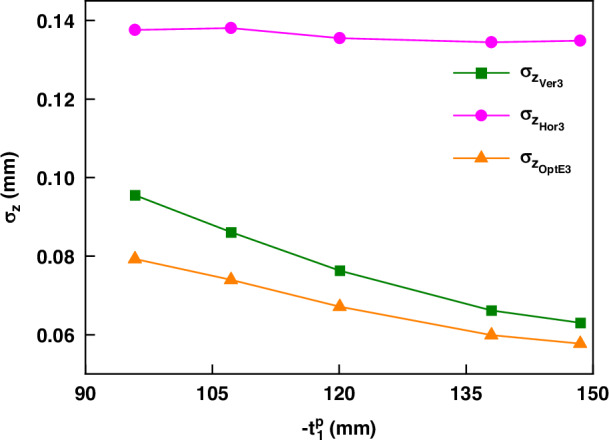
The precision result measured for FPP systems (#12-#16) in different ${t}_{1}^{p}$

We have discussed in point (ii) after Eq. (17) that ${B}_{e2}$ is independent of ${t}_{1}^{p}$. Although this is somewhat intuitive, it has not been explicitly mentioned in literature. Thus, validation is necessary for confirmation. We constructed additional five FPP systems (FPP #12-#16), where we aimed to keep ${t}_{2}^{p}$ as constant as possible while purposely changed ${t}_{1}^{p}$. Figure 8 shows the measurement precision of different methods with varying ${t}_{1}^{p}$, in which the precision of Hor3 indeed remains nearly constant, while that of Ver3 and OptE3 increase with the increase in ${t}_{1}^{p}$. Table 4 more clearly presents the values of ${t}_{1}^{p}$, ${t}_{2}^{p}$ and the precision in these five FPP systems. The verification of the general Pythagorean relationship in Eq. (7) is also consistent with the theoretical results.

**Table 4:** Experimental Results for FPP systems (#12-#16)

Systems	${t}_{1}^{p}$/mm	${t}_{2}^{p}$/mm	${\sigma }_{{z}_{{Ver}3}}$/mm	${\sigma }_{{z}_{{Hor}3}}$/mm	${\sigma }_{{z}_{{OptE}3}}$/mm	Q
#12	−95.880	−66.574	0.096	0.138	0.079	1.003
#13	−107.213	−67.105	0.086	0.138	0.074	1.014
#14	−120.081	−66.960	0.076	0.136	0.067	1.010
#15	−138.008	−67.568	0.066	0.135	0.060	1.012
#16	−148.522	−67.770	0.063	0.135	0.058	1.016

### B4. Validation of the angle between the optical axes

The angle between the optical axes was derived into Eq. (20), i.e., $\cos \left(\omega \right)={r}_{33}^{p}$. As we did not see this result in the FPP literature, we provide an intuitive validation by the following experiment.

First, we construct yet another FPP system (FPP #17) and calibrate its intrinsic and extrinsic parameters, from which, we obtain ${r}_{33}^{p}=0.987$. According to our theoretical result in Eq. (20), $\omega ={\cos }^{-1}\left({r}_{33}^{p}\right)={9.25}^{^\circ }$.

**Fig. 9:**
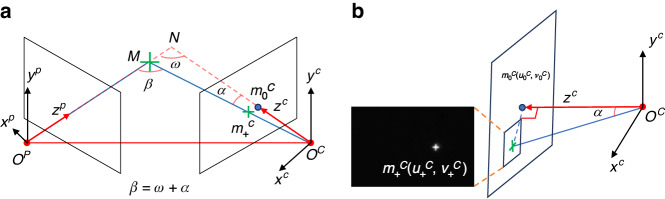
The measurement principle for the angle $\omega$. **a** Imaging process of cross center and the angle relationship diagram. **b** Measurement principle of the angle $\alpha$

Second, we independently measure this angle. As shown in Fig. 9a, we generated a cross pattern, with the center of the cross corresponding to the integer pixel position closest to the projector’s principal point. The projector projects this pattern onto a white flat plate, where it forms a clear image. The camera captures the cross-pattern image on the plate. A triangle, ${O}^{c}M{O}^{P}$, is formed as shown in Fig. 9a, whose edges are measured as $\left|M{O}^{c}\right|=770\,{mm}$, $\left|M{O}^{p}\right|=720\,{mm}$, and $\left|{{O}^{p}O}^{c}\right|=165\,{mm}$. Using the law of cosines, we have $\omega {\prime} =\beta ={12.12}^{^\circ }$.

Third, by comparing the calibrated and measured results, there is a difference of ${\omega {\prime} -\omega =2.87}^{^\circ }$. This comes from the fact that $M{O}^{c}$ is generally not the optical axis of the camera, i.e., usually the optical axes of the projector and the camera are skew lines and do not intersect. We thus further measured the angle $\alpha$ for compensation, as shown in Fig. 9b. In the triangle ${m}_{0}^{c}{O}^{c}{m}_{+}^{c}$,

50 $$\tan \left(\alpha \right)=\frac{\sqrt{{\left({{u}_{+}^{c}-u}_{0}^{c}\right)}^{2}+{\left({{v}_{+}^{c}-v}_{0}^{c}\right)}^{2}}}{{f}_{u}^{c}}$$

where $\left({u}_{0}^{c},{v}_{0}^{c}\right)$ and ${f}_{u}^{c}$ have been calibrated to be (1198.505,1039.370) and $7299.880$, while $\left({u}_{+}^{c},{v}_{+}^{c}\right)$ is measured to be (1617,1075). We obtain $\alpha$ to be ${3.29}^{^\circ }$. We compensate the angle as $\omega {\prime} {\prime} \approx \beta -\alpha ={8.83}^{^\circ }$, with the absolute error reduced to ${\omega }^{{\prime} {\prime} }-\omega ={0.42}^{^\circ }$, and a relative error of 4.5%, which verifies Eq. (20). As mentioned earlier, the optical axes of the camera and projector do not intersect, but we have assumed that they intersect here. We thus emphasize that it is merely an estimation.

### B5. Validation of the precision model and FPP system design software

We first validate the SUPM in Eq. (21). In addition, we present a case study of FPP system design guided by the FPP-Planner software developed in this work. According to ref. ^22^, the camera employed in our experiments has a saturation capacity of ${\mu }_{e.{Sat}}=10345{e}^{-}$ and a gain of $K=0.0232\,{DN}{\left({e}^{-}\right)}^{-1}$.

**Fig. 10:**
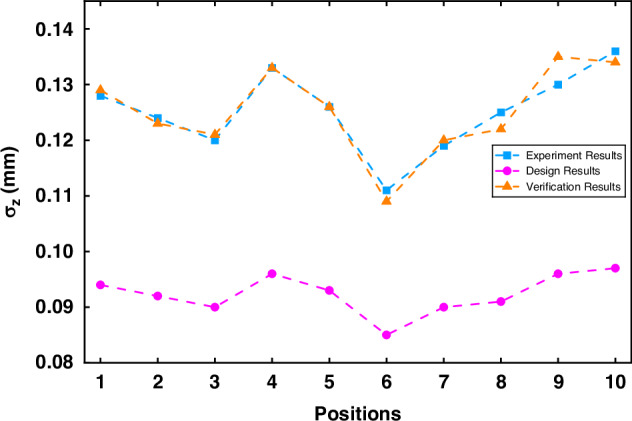
Validation results of the practical model against experimental data

We verify Eq. (21) for FPP #1-#10 through the following procedure: (i) substituting *N* = 3 and ${\mu }_{e.{Sat}}$ into Eq. (23) to calculate the phase standard deviation; (ii) substituting the calculated phase standard deviation, each object-plane position ${z}^{w}$, the fringe period *T*, and the calibration parameters from Eqs. (45–46) into Eq. (21). The results are shown in Fig. 10, where the magenta data represent the theoretically designed system precision, while the blue data correspond to the experimentally measured precision of FPP #1.

The discrepancy between designed and measured precision is observed, as expected. It arises from the fact that Eq. (24) provides the theoretical limit of the phase standard deviation in the ideal situation that ${A}^{c}={B}^{c}={I}_{{sat}}/2$, where ${A}^{c}$ and ${B}^{c}$ are the background and the modulation intensity of the fringes and ${I}_{{sat}}$ is the saturation intensity. Indeed, the theoretical precision limit is shown to be better than the experimental one, which provides a good reference value of the precision limit during the FPP design phase.

However, in practice, it is often seen that ${B}^{c}$ < ${I}_{{sat}}$ due to object surface property, and ${B}^{c}/{A}^{c} < 1$ due to the modulation transfer function (MTF)^48^ of the optical system, especially for high-frequency fringe patterns. For example, ${B}^{c}/{A}^{c}$ measured is 0.82 in our experiments. Therefore, the following model is a better option for evaluation after the system has been set up^22^,

51 $${\sigma }_{\varphi }^{2}=\frac{2}{{\left({B}^{c}\right)}^{2}}K{A}^{c}$$

After substituting Eq. (51) into Eq. (21), we obtained our theoretical evaluation results as shown in yellow, which present an excellent agreement with the experimental data, with the maximum relative error across all results being only 4%.

To summarize, in the above validation, the excellent agreement between the verification results (yellow) and the experiment results (blue) shows the validity of the SUPM, while the design result with a high precision (magenta) serves a good reference target. This motivates us to further prototype an FPP-planner software to bridge theory and practice based on the SUPM.

According to the design strategies summarized in Table 1, when ${f}_{u}^{p}\ne {f}_{v}^{p}$ (case I), it has been indicated that $\left[{\left({B}_{e}\right)}_{M},\omega ,N,{\mu }_{e.{Sat}}\right]$ will be the designable variables. To increase the design flexibility, ${\sigma }_{{zM}},z$ and $T$ are also included. In fact, ${\sigma }_{{zM}}$ is the targeted precision of FPP, which is set by the user according to application requirements; $z$ denotes the distance from the optical component (camera or projector, depending on the chosen strategy) to the object, which is often determined by practical requirement but may also be a free parameter; although $T$ can be pre-determined as mentioned earlier, we keep it as a designable parameter. This expands the designable parameters into seven, i.e., $\left[{\left({B}_{e}\right)}_{M},\omega ,N,{\mu }_{e.{Sat}},{\sigma }_{{zM}},z,T\right]$. As for ${f}_{u}^{p}\approx {f}_{v}^{p}=f$ (case II), there will be nine designable parameters as $\left[{\left(B\right)}_{M},\omega ,N,{\mu }_{e.{Sat}},{\sigma }_{{zM}},z,T,F,\delta \right]$, where the additional two parameters $F$ (physical focal length) and $\delta$ (the pixel size of the projector) are used to determine the focal length $f$ so that $f=F/\delta$. Although two more parameters are added, the design is more physically intuitive. In our current FPP-Planner software, instead of randomly assigning the values of all parameters in a trial-and-error manner, we let the software to compute and recommend $\left[{\left({B}_{e}\right)}_{M},\omega \right]$ (or $\left[{\left(B\right)}_{M},\omega \right]$) while the rest of parameters are decided by the user.

Finally, we use our FPP-Planner to design a system under Strategy I and Case II with a targeted precision better than 50 $\mu m$. Our design considerations are as follows:

(i) To account for the approximations in model derivation and other practical factors in a non-ideal environment, as mentioned in Discussions, in order to achieve the targeted precision (TP), we set a more stringent precision requirement during the design phase, which is called design-adjusted precision (DAP). We set a higher precision requirement as ${\sigma}_{{z}\_{DAP}}={35}{\mu}{m}$. For a less experienced user, it is recommended to set ${{\sigma}}_{z}\_{DAP}={\sigma}_{z}\_{TP}/{2}$.
(ii) In order to easily validate our design later, we rely on our currently available laboratory hardware which has been used in the earlier experiments. We set ${{z}}^{{w}}$ = 840 mm. The following parameters can be found directly: the saturation capacity of the camera is ${{\mu}}_{{e}.{Sat}}={10},$345 e^−^, the projector focal length is *f*$={25}$ *mm*, and the pixel size of the projector is *δ* = 7.56 *μm*. In addition, the fringe period is set at *T* = 21^22^;
(iii) The number of phase-shifting steps *N* varied from 3 to 9, in order to provide more possible design recommendations;
(iv) With the above settings, possible combinations of $\left[{\left({B}\right)}_{{M}},{\omega}\right]$ will be the output of the FPP-planner for users to choose.

**Fig. 11:**
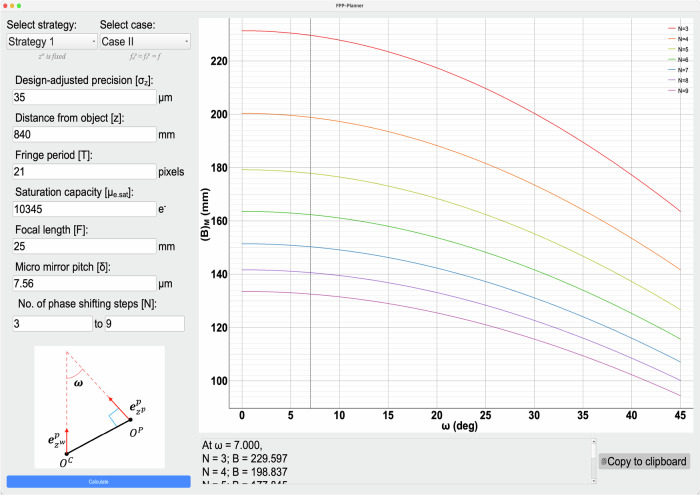
FPP-Planner

Figure 11 presents the recommended parameter pairs, $\left[{\left(B\right)}_{M},\omega \right]$, for different values of *N*, which are obtained from Eq. (21) by using the above parameter values and the DAP requirements. Among many recommended design solutions, we focus on *N* = 3 since it requires the least number of fringe patterns for projection. We then choose one particular solution with $\left[{\left(B\right)}_{M},\omega \right]\,$= $\left[230\,{mm},7^\circ \right]$ which is easy to configure. We indeed constructed an FPP system, FPP #18, accordingly, with a measured baseline of 235 mm and a measured angle of 7.7°. Experimental measurements are carried out by measuring the plane target at 10 different locations. The experimental procedure is similar to the one yielding Fig. 10. Our newly constructed FPP system demonstrates a precision of $49.5\,\mu m$, which fulfills our design goal.

## Supplementary information

Supplementary information

## Data Availability

Data underlying the results presented in this paper are not publicly available at this time but may be obtained from the authors upon reasonable request.
