# Supplementary material for: On the precision models of fringe projection profilometry: unification, simplification and connection
Source: Light Sci Appl. 2026 May 12;15:232. doi: 10.1038/s41377-026-02300-x (PMC13168703; doi:10.1038/s41377-026-02300-x)
Supplement: Supplementary file 1 — Supplementary information [file 41377_2026_2300_MOESM1_ESM.docx]

Supplementary information for

On the precision models of fringe projection profilometry: unification, simplification and connection

Shenzhen Lv^1^, Nengqi Huang^1^, Yuxuan Zou^1^, Chengeng Liu^1^, Siah Yee Long^1^, Dawei Tang^2^, Xiangqian Jiang^2^ and Qian Kemao^1,*^

^1^College of Computing and Data Science, Nanyang Technological University, 639798, Singapore

^2^Centre for Precision Technologies, University of Huddersfield, Huddersfield HD1 3DH, UK

^*^Correspondence to Qian Kemao: [mkmqian@ntu.edu.sg](mailto:mkmqian@ntu.edu.sg)

**Supplementary Notes**

1. **The pinhole model and the epipolar line**

Referring to Fig. 1, the pinhole model of a camera relates a 3D object point in a world coordinate system, $\left( x^{w},y^{w},z^{w} \right)$, and its image point in the image coordinate system, $\left( u^{c},v^{c} \right)$, as follows [1],

$$s^{c}\left[ u^{c}, v^{c},1 \right]^{t}=A^{c}\left[ \begin{matrix} R^{c} & \mathbf{t}^{c} \end{matrix} \right]\left[ x^{w},y^{w},z^{w},1 \right]^{t} (A1)$$

where the superscript *c* refers to the camera; $s^{c}$ is an arbitrary scale factor; $A^{c}=\left[ \begin{matrix} f_{u}^{c} & 0 & u_{0}^{c} \\ 0 & f_{v}^{c} & v_{0}^{c} \\ 0 & 0 & 1 \end{matrix} \right]$ is the intrinsic matrix, with $f_{u}^{c}$ and $f_{v}^{c}$ being the respective focal lengths of the camera lens along $u^{c}$ and $v^{c}$ directions, respectively, and $\left( u_{0}^{c}, v_{0}^{c} \right)$ being the camera’s principle point coordinate; $R^{c}=\left[ r_{ij}^{c} \right]_{3\times3}$ is a rotation matrix; $\mathbf{t}^{c}=\left[ t_{j}^{c} \right]_{\mathbf{3}\times\mathbf{1}}=\left[ t_{1}^{c},t_{2}^{c},t_{3}^{c} \right]^{t}$ is a translation vector; the superscript *t* represents the transpose operation. Note that there is another important coordinate system called camera coordinate system (CCS), where the origin is the optical center, the *x* and *y* axes are parallel to those in the image coordinate system, while the *z* axis is the optical axis. We now select CCS as the world coordinate system so that $R^{c}$ is an identity matrix and $\mathbf{t}^{c}$ is a zero vector, which will greatly simplify our analysis. Substituting the special $R^{c}$ and $\mathbf{t}^{c}$ into Eq. (A1) gives

$$x^{w}=\frac{u^{c}-u_{0}^{c}}{f_{u}^{c}}z^{w} (A2)$$

$$y^{w}=\frac{v^{c}-v_{0}^{c}}{f_{v}^{c}}z^{w} (A3)$$

Similarly, the pinhole model of a projector is expressed as [2]

$$s^{p}\left[ u^{p}, v^{p},1 \right]^{t}=A^{p}\left[ \begin{matrix} R^{p} & \mathbf{t}^{p} \end{matrix} \right]\left[ x^{w},y^{w},z^{w},1 \right]^{t} (A4)$$

where the superscript $p$ emphasizes that the parameters are related to the projector, while the other symbols carry the same meaning as for the camera parameters in Eq. (A1). By canceling the unknown scalar $s^{p}$ in Eq. (A4), the following two equations are obtained,

$$u^{p}=\frac{\left[ \left( f_{u}^{p}r_{11}^{p}+r_{31}^{p}u_{0}^{p} \right)x^{w}+\left( f_{u}^{p}r_{12}^{p}+r_{32}^{p}u_{0}^{p} \right)y^{w}+\left( f_{u}^{p}r_{13}^{p}+r_{33}^{p}u_{0}^{p} \right)z^{w}+\left( f_{u}^{p}t_{1}^{p}+t_{3}^{p}u_{0}^{p} \right) \right]}{r_{31}^{p}x^{w}+r_{32}^{p}y^{w}+r_{33}^{p}z^{w}+t_{3}^{p}} (A5)$$

$$v^{p}=\frac{\left[ \left( f_{v}^{p}r_{21}^{p}+r_{31}^{p}v_{0}^{p} \right)x^{w}+\left( f_{v}^{p}r_{22}^{p}+r_{32}^{p}v_{0}^{p} \right)y^{w}+\left( f_{v}^{p}r_{23}^{p}+r_{33}^{p}v_{0}^{p} \right)z^{w}+\left( f_{v}^{p}t_{2}^{p}+t_{3}^{p}v_{0}^{p} \right) \right]}{r_{31}^{p}x^{w}+r_{32}^{p}y^{w}+r_{33}^{p}z^{w}+t_{3}^{p}} (A6)$$

Accordingly, the epipolar line has also been obtained as [3]

$$u^{p}l_{1}+v^{p}l_{2}+l_{3}=0 (A7)$$

where

$l_{1}=\frac{1}{f_{u}^{c}f_{u}^{p}}\left[ \left( u^{c}-u_{0}^{c} \right)\left( -r_{21}^{p}t_{3}^{p}+t_{2}^{p}r_{31}^{p} \right)+\left( v^{c}-v_{0}^{c} \right)\left( t_{2}^{p}r_{32}^{p}-r_{22}^{p}t_{3}^{p} \right)\frac{f_{u}^{c}}{f_{v}^{c}}+\left( -r_{23}^{p}t_{3}^{p}+t_{2}^{p}r_{33}^{p} \right)f_{u}^{c} \right] (A8)$

$l_{2}=\frac{1}{f_{v}^{p}f_{u}^{c}}\left[ \left( u^{c}-u_{0}^{c} \right)\left( r_{11}^{p}t_{3}^{p}-t_{1}^{p}r_{31}^{p} \right)+\left( v^{c}-v_{0}^{c} \right)\left( r_{12}^{p}t_{3}^{p}-t_{1}^{p}r_{32}^{p} \right)\frac{f_{u}^{c}}{f_{v}^{c}}+\left( r_{13}^{p}t_{3}^{p}-t_{1}^{p}r_{33}^{p} \right)f_{u}^{c} \right] (A9)$

$$l_{3}=\frac{\left( u^{c}-u_{0}^{c} \right)}{f_{u}^{c}}\left[ \frac{u_{0}^{p}}{f_{u}^{p}}\left( r_{21}^{p}t_{3}^{p}-t_{2}^{p}r_{31}^{p} \right)+\frac{v_{0}^{p}}{f_{v}^{p}}\left( -r_{11}^{p}t_{3}^{p}+t_{1}^{p}r_{31}^{p} \right)-t_{2}^{p}r_{11}^{p}+t_{1}^{p}r_{21}^{p} \right]$$

$$+\frac{\left( v^{c}-v_{0}^{c} \right)}{f_{v}^{c}}\left[ \frac{u_{0}^{p}}{f_{u}^{p}}\left( r_{22}^{p}t_{3}^{p}-r_{32}^{p}t_{2}^{p} \right)+\frac{v_{0}^{p}}{f_{v}^{p}}\left( -r_{12}^{p}t_{3}^{p}+t_{1}^{p}r_{32}^{p} \right)-t_{2}^{p}r_{12}^{p}+t_{1}^{p}r_{22}^{p} \right]$$

$$+\left[ \frac{u_{0}^{p}}{f_{u}^{p}}\left( r_{23}^{p}t_{3}^{p}-r_{33}^{p}t_{2}^{p} \right)+\frac{v_{0}^{p}}{f_{v}^{p}}\left( t_{1}^{p}r_{33}^{p}-r_{13}^{p}t_{3}^{p} \right)+\left( -t_{2}^{p}r_{13}^{p}+t_{1}^{p}r_{23}^{p} \right) \right] (A10)$$

Furthermore, we have theoretically derived that the optimal angle direction is perpendicular to the direction of the epipolar line [4], i.e.,

$$\tan\left( \theta_{opt} \right)=-\frac{l_{2}}{l_{1}}=\frac{f_{u}^{p}\left[ \begin{aligned} \left( r_{11}^{p}t_{3}^{p}-t_{1}^{p}r_{31}^{p} \right)\frac{\left( u^{c}-u_{0}^{c} \right)}{f_{u}^{c}}+\left( r_{12}^{p}t_{3}^{p}-t_{1}^{p}r_{32}^{p} \right)\frac{\left( v^{c}-v_{0}^{c} \right)}{f_{v}^{c}} \\ +\left( r_{13}^{p}t_{3}^{p}-t_{1}^{p}r_{33}^{p} \right) \end{aligned} \right]}{f_{v}^{p}\left[ \begin{aligned} \left( r_{21}^{p}t_{3}^{p}-t_{2}^{p}r_{31}^{p} \right)\frac{\left( u^{c}-u_{0}^{c} \right)}{f_{u}^{c}}+\left( r_{22}^{p}t_{3}^{p}-t_{2}^{p}r_{32}^{p} \right)\frac{\left( v^{c}-v_{0}^{c} \right)}{f_{v}^{c}} \\ +\left( r_{23}^{p}t_{3}^{p}-t_{2}^{p}r_{33}^{p} \right) \end{aligned} \right]} (A11)$$

where $\theta_{opt}\left[ 0,\pi\right]$ is the angle between the fringe orientation and the horizontal axis. This property reveals the interesting and important presence of the epipolar line in FPP.

1. **The FPP workflow**

With the above knowledge, the Ver3/Hor3/OptE3 methods are described step by step as follows, which are correspondingly illustrated in Supplementary Note Fig. 1:

1. Calibration: The system calibration is needed to find the intrinsic and extrinsic parameters on the right side of the Eqs. (A1) and (A4);
2. Fringe projection and phase calculation: Fringe patterns will be projected by the projector and captured by the camera, where Ver3/Hor3/OptE3 project vertical, horizontal and optimal-angle fringe patterns, respectively. By using a phase measurement method and a phase unwrapping method, the absolute phase value of any camera pixel $\left( u^{c},v^{c} \right)$ can be computed;
3. Pixel correspondence: For each camera pixel $\left( u^{c},v^{c} \right)$, its corresponding projector pixel $\left( u^{p},v^{p} \right)$ is searched in order to form a pair, based on the fact that they share the same phase. Each pair will later be used to reconstruct an object point $\left( x^{w},y^{w},z^{w} \right)$. An interesting problem is that the pixel correspondence is not one-to-one. For the phase value of $\left( u^{c},v^{c} \right)$, there are many projector pixels having the same value, locating on an equal-phase line. A simple but smart idea is to project vertical fringes so that the equal-phase line is also vertical, from which $u^{p}$ can be uniquely determined although $v^{p}$ remains unknown. Interestingly, three equations based on $u^{c}$, $v^{c}$ and $u^{p}$ are sufficient to reconstruct $\left( x^{w},y^{w},z^{w} \right)$. This is the main idea of Ver3. This smart idea can be extended to using horizontal fringe patterns and searching for $v^{p}$, which becomes Hor3. On the contrary, OptE3’s fringe orientation, as well as the equal-phase line, is generally slanted and does not have the convenience in Ver3/Hor3. However, since $\left( u^{p},v^{p} \right)$ is also located on the epipolar line, which is known after calibration, it can be easily determined as the intersection point of the equal-phase line and the epipolar line [4];
4. Reconstruction: based on the pixel correspondence, the reconstruction becomes straightforward. For Ver3, with the obtained correspondence between $\left( u^{c},v^{c} \right)$ and $u^{p}$, three equations, Eq. (A2) based on $u^{c}$, Eq. (A3) based on $v^{c}$ and Eq. (A5) based on $u^{p}$, are used for 3D reconstruction. Similarly, for Hor3, with the correspondence between $\left( u^{c},v^{c} \right)$ and $v^{p}$, Eqs. (A2), (A3) and (A6) are used for 3D reconstruction. For OptE3, since the correspondence between $\left( u^{c},v^{c} \right)$ and $\left( u^{p},v^{p} \right)$ is achieved, four equations, (A2), (A3), (A5) and (A6), can be used for a more precise reconstruction. More interestingly, we have proved that one equation among the four is redundant because the earlier involvement of the epipolar constraint in the correspondence step [4]. Therefore, either Eqs. (A2), (A3) and (A5), or Eqs. (A2), (A3) and (A6), can be used for 3D reconstruction.

Among the above four steps, the calibration is common for Ver3/Hor3/OptE3, except that OptE3 also calculates the epipolar line, which is readily; the phase calculation component is also common for Ver3/Hor3/OptE3. Thus, this paper will focus on the fringe directions, the correspondence step and the reconstruction step.


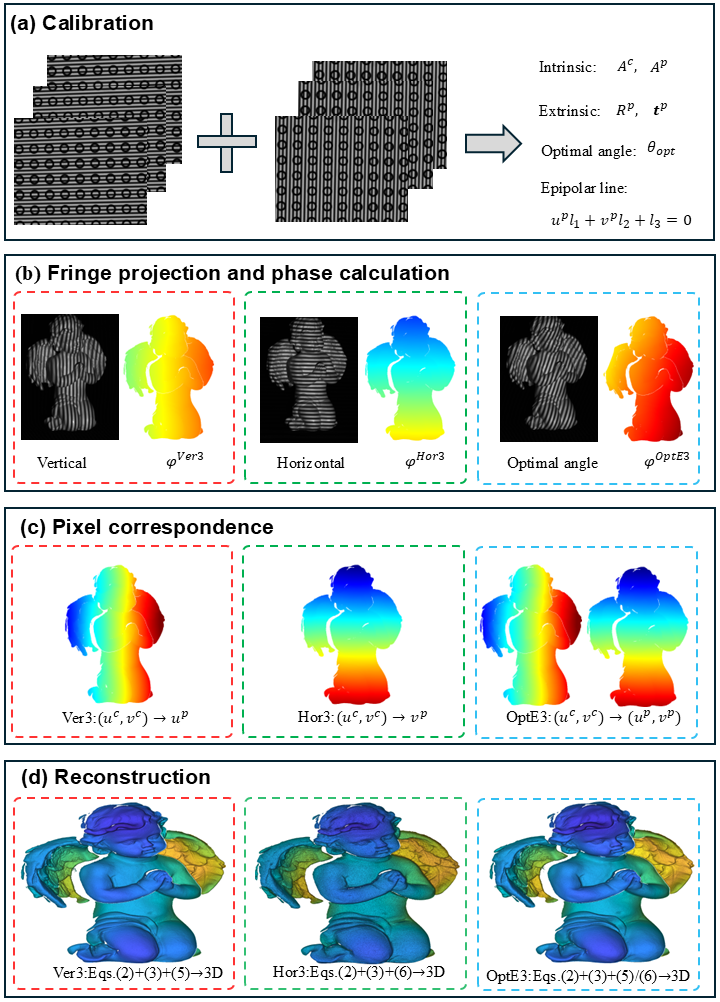


**Supplementary Note Fig. 1. The workflow of Ver3/Hor3/OptE3 .** (a) Calibration of camera and projector to obtain their intrinsic and extrinsic paramters. Optimal angle and epipolar line can also be obtained from the calibrrated paramters, which are used for OptE3; Illustraed here is one of the calibration methods [2]; (b) From left to right, we use red, green and blue boxes to indicate Ver3, Hor3 and OptE3 methods, which applies to (c) and (d) as well, so that while the workflow is explained, the main simialities and differences are also highlighted. After phase-shifted fringe patterns (with different angles for different methods) are projcted and captured, phase can be obtained through phase-shifting and phase unwrapping; (c) Based on the obtained phase value, a project pixel and a camera pixel can be corresponded. Fox example, in the red box, for all the camera pixels, their correspoinding $u^{p}$ coordinates of the projector are calcualted and displayed; (d) The corresponded pixels are used for 3D reconstruction. Hor3 (in green box) can be observed to be inferior, while Ver3 (in red box) and OptE3 (in blue box) are almost identical by naked eyes. This is because the FPP system is manually adjusted to satisfy $t_{1}^{p}\approx3t_{2}^{p}$(see the dicussion in point (iv) below Eq. (17)). However, their difference will incerese depending on the magnitude of the effective baseline (See B1 of Materials and Methods.Validation of the constant optimal angle, FPP #1). Making precision quantifiable and predicable is the main theme of this paper. Although our experiemental results of an Angel statue is used for illustration of the workflow, a standard plane target is extensively used in this paper for quantifying our proposed precision models.

1. **The theoretical error introduced by removing FOV-related terms**

Removing the FOV terms corresponds to neglecting the first two terms in Eq. (8). We therefore define the relative error $E_{r}$ as,

$$Er=\left| \frac{r_{31}^{p}\frac{\left( u^{c}-u_{0}^{c} \right)}{f_{u}^{c}}+r_{32}^{p}\frac{\left( v^{c}-v_{0}^{c} \right)}{f_{v}^{c}}}{r_{33}^{p}} \right| (C1)$$

To estimate the range of $Er$, we note the following facts:
(i) $(u^{c}-u_{0}^{c})/f_{u}^{c}$ and $(v^{c}-v_{0}^{c})/f_{v}^{c}$ represent the tangents of the half-FOV along the horizontal and vertical directions of the camera, respectively. Without loss of generality, we assume that the horizontal and vertical FOVs are identical, denoted as FOV;
(ii) $r_{33}^{p}=\cos(\omega)$, where $\omega$is the angle between the camera and projector optical axes;
(iii) the orthonormality of the rotation matrix $\mathbf{R}$ gives

$$\left( r_{31}^{p} \right)^{2}+\left( r_{32}^{p} \right)^{2}+\left( r_{33}^{p} \right)^{2}=1 (C2)$$

With the first two facts above, Eq. (C1) can be re-written as:

$$Er=\left| \frac{r_{31}^{p}+r_{32}^{p}}{r_{33}^{p}} \right|\times tan\left( {FOV}/2 \right) (C3)$$

By using the Cauchy–Schwarz inequality, and the third fact, we have

$$\left| r_{31}^{p}+r_{32}^{p} \right|\leq\sqrt{2}\sqrt{\left[ \left( r_{31}^{p} \right)^{2}+\left( r_{32}^{p} \right)^{2} \right]}=\sqrt{2}sin\left( \omega\right) (C4)$$

Combining Eq. (C3) and the inequality (C4) yields

$$Er\leq\sqrt{2}\tan\left( \frac{FOV}{2} \right)\tan\left( \omega\right) (C5)$$

For typical FPP systems, the optical-axis angle $\omega$ is usually smaller than ${10}^{\circ}$. When $\mathrm{FOV}={22.9}^{\circ}$, we have $Er\leq5\%$.

**References**

1. Z. Zhang, "A flexible new technique for camera calibration," IEEE Transactions on Pattern Analysis and Machine Intelligence **22**, 1330–1334 (2000).

2. Zhang, S. & Huang, P. S. Novel method for structured light system calibration. *Optical Engineering* **45**, 083601 (2006).

3. Hartley, R. & Zisserman, A. Multiple View Geometry in Computer Vision. 2nd edn. (Cambridge: Cambridge University Press, 2003).

4. Lv, S. Z. et al. Fringe projection profilometry method with high efficiency, precision, and convenience: theoretical analysis and development. *Optics Express* **30**, 33515-33537 (2022).
